# Lytic polysaccharide monooxygenases and other oxidative enzymes are abundantly secreted by *Aspergillus nidulans* grown on different starches

**DOI:** 10.1186/s13068-016-0604-0

**Published:** 2016-09-01

**Authors:** Laura Nekiunaite, Magnus Ø. Arntzen, Birte Svensson, Gustav Vaaje-Kolstad, Maher Abou Hachem

**Affiliations:** 1 Enzyme and Protein Chemistry, Department of Biotechnology and Biomedicine, Technical University of Denmark, Elektrovej 375, 2800 Kgs Lyngby, Denmark; 2 Department of Chemistry, Biotechnology and Food Science, Norwegian University of Life Sciences, PO Box 5003, 1430 Ås, Norway

**Keywords:** *Aspergillus nidulans*, Biofuels, Carbohydrate-active enzymes, Carbohydrate-binding module family 20 (CBM20), Filamentous fungi, Lytic polysaccharide monooxygenase (LPMO), Starch, Starch-binding

## Abstract

**Background:**

Starch is the second most abundant plant-derived biomass and a major feedstock in non-food industrial applications and first generation biofuel production. In contrast to lignocellulose, detailed insight into fungal degradation of starch is currently lacking. This study explores the secretomes of *Aspergillus nidulans* grown on cereal starches from wheat and high-amylose (HA) maize, as well as legume starch from pea for 5 days.

**Results:**

*Aspergillus nidulans* grew efficiently on cereal starches, whereas growth on pea starch was poor. The secretomes at days 3–5 were starch-type dependent as also reflected by amylolytic activity measurements. Nearly half of the 312 proteins in the secretomes were carbohydrate-active enzymes (CAZymes), mostly glycoside hydrolases (GHs) and oxidative auxiliary activities (AAs). The abundance of the GH13 α-amylase (AmyB) decreased with time, as opposed to other starch-degrading enzymes, e.g., the GH13 AmyF, GH15 glucoamylases (GlaA and GlaB), and the GH31 α-glucosidase (AgdE). Two AA13 LPMOs displayed similar secretion patterns as amylolytic hydrolases and were among the most abundant CAZymes. The starch-active *An*LPMO13A that possesses a CBM20 carbohydrate-binding module dominated the starch-binding secretome fraction. A striking observation is the co-secretion of several redox-active enzymes with the starch-active AA13 LPMOs and GHs, some at high abundance. Notably nine AA9 LPMOs, six AA3 sub-family 2 (AA_2) oxidoreductases, and ten AA7 glyco-oligosaccharide oxidases were identified in the secretomes in addition to other non-CAZyme oxidoreductases.

**Conclusions:**

The co-secretion and high abundance of AA13 LPMOs are indicative of a key role in starch granule deconstruction. The increase in AA13 LPMO abundance with culture time may reflect accumulation of a more resistant starch fraction towards the later stages of the culture. The identification of AmyR sites upstream AA13 LPMOs unveils co-regulation of LPMOs featuring in starch utilization. Differential deployment of amylolytic hydrolases and LPMOs over time suggests additional regulatory mechanisms. The abundant co-secretion of distinct AA3 and AA7 oxidoreductases merits further studies into their roles and possible interplay with LPMOs and other enzymes in the deconstruction of starchy substrates. The study reports for the first time the biological significance of LPMOs in starch degradation and the temporal interplay between these and amylolytic hydrolases.

**Electronic supplementary material:**

The online version of this article (doi:10.1186/s13068-016-0604-0) contains supplementary material, which is available to authorized users.

## Background

Starch is one of the most abundant renewable biopolymers in nature [1]. Annually two billion tons of starch crops are harvested worldwide, making it an attractive resource for industrial applications such as production of first generation biofuels, pharmaceuticals, textiles, detergents, paper, and food [1]. Starch consists of the two α-glucan polymers, amylose and amylopectin. Amylose is an essentially linear polymer of α-(1,4)-linked glucosyl units, while amylopectin which constitutes 70–80 % of starch granules, is a branched macromolecule having α-(1,4)-glucan chains branched with approximately 5 % α-(1,6)-glucosidic linkages [2]. Despite this chemical simplicity, the α-glucan chains are arranged radially in a supramolecular assembly forming water insoluble granules varying in size (1–150 µm), morphology, crystal-type packing, and crystallinity (15–45 %) [3, 4]. This organization renders starch, especially its crystalline regions, into a relatively challenging substrate for complete enzymatic deconstruction. The extent of starch resistance to enzymatic hydrolysis is correlated to botanical origin and processing, both factors having influence on crystal packing, crystallinity, and supramolecular structure of the starch granule.

The high utilization potential of starch as a renewable biological resource and an industrial feedstock motivates efforts to improve starch hydrolysis yields, particularly from more resistant starch types and for shortening process times. Gain in yields of hydrolysis would have a considerable impact on efficiency and cost of industrial starch processing as well as the reduction of environmental impact of this process [5].

The classical paradigm of polysaccharide degradation by hydrolytic enzymes, which has been valid for decades, was recently re-visited by the discovery of the copper-dependent lytic polysaccharide monooxygenases (LPMOs) [6, 7]. LPMOs catalyze the oxidative cleavage of glycosidic linkages of polysaccharides in the presence of molecular oxygen and an external electron donor, by hydroxylation of either the C1 or C4 carbon of the glycosidic bond [8, 9]. These enzymes play an instrumental role in degradation of recalcitrant crystalline polysaccharides such as cellulose [7, 10] and chitin [6], which renders LPMOs into pivotal tools in industrial biomass conversion [11]. Recently it has been established that some LPMOs oxidize non-crystalline hemicelluloses and soluble cello-oligosaccharides [12–15]. Moreover, the recent discovery of starch-active LPMOs [16–18], assigned into auxiliary activity family 13 (AA13) in the CAZy database [19], suggest that LPMOs play a role in starch degradation together with amylolytic hydrolases.

Filamentous fungi secrete impressive amounts of hydrolytic enzymes targeting polysaccharides. In addition to glycoside hydrolyses (GHs), several fungi also produce a multitude of LPMOs [20, 21]. In contrast to lignocellulose matrices, little is known regarding the type and composition of enzyme cocktails deployed by fungi for the degradation of starches differing in botanical origin and properties. Notably, the involvement of oxidative enzymes including LPMOs in starch degradation has not been demonstrated in vivo. Here, we provide new insight into the excellent starch-degrading capabilities of the well-studied saprophytic ascomycete *Aspergillus nidulans* that is taxonomically related to well-established industrial cell factory species such as *Aspergillus niger* and *Aspergillus oryzae* [22]. By integrating secretomics and enzyme activity assays, we analyzed temporal changes of the enzymes secreted by *A. nidulans* to sustain growth on three different starches in the course of 5 days. The data demonstrate differences in growth and secretomes on the selected starch substrates. A common feature of growth on starch was that two AA13 LPMOs including the modular starch-specific enzyme joint to a starch-binding domain of family 20 (CBM20) were among the most abundant CAZymes together with a variety of LPMOs and other oxidative enzymes. This finding suggests that oxidative cleavage of α-glucosidic bonds plays a significant role in starch breakdown. Altogether, the novel insight into enzymatic activities secreted by *A. nidulans* and related fungi for efficient starch breakdown is relevant for design of enzyme mixtures with enhanced bioconversion efficiencies of starches especially those resistant to hydrolytic degradation.

## Results

### Starch substrates and fungal growth

To assess the ability of *A. nidulans* to sense differences in the origin and structure of the starch substrates and to fine-tune the composition of secreted enzymes accordingly, this fungus was grown on wheat, high-amylose (HA) maize, and pea starches, and the resulting secretomes were analyzed. *Aspergillus nidulans* grew efficiently on wheat and HA maize starch and no intact starch granules were distinguished from the fungal biomass after 5 days, suggesting extensive degradation of both starches. By contrast, growth was poor on pea starch, leaving significant amounts of intact starch granules at culture harvest, which clearly demonstrates important differences due to the botanical origin and properties of the starch on enzymatic deconstruction and growth.

### Enzymatic analysis of amylolytic activities

**Fig. 1:**
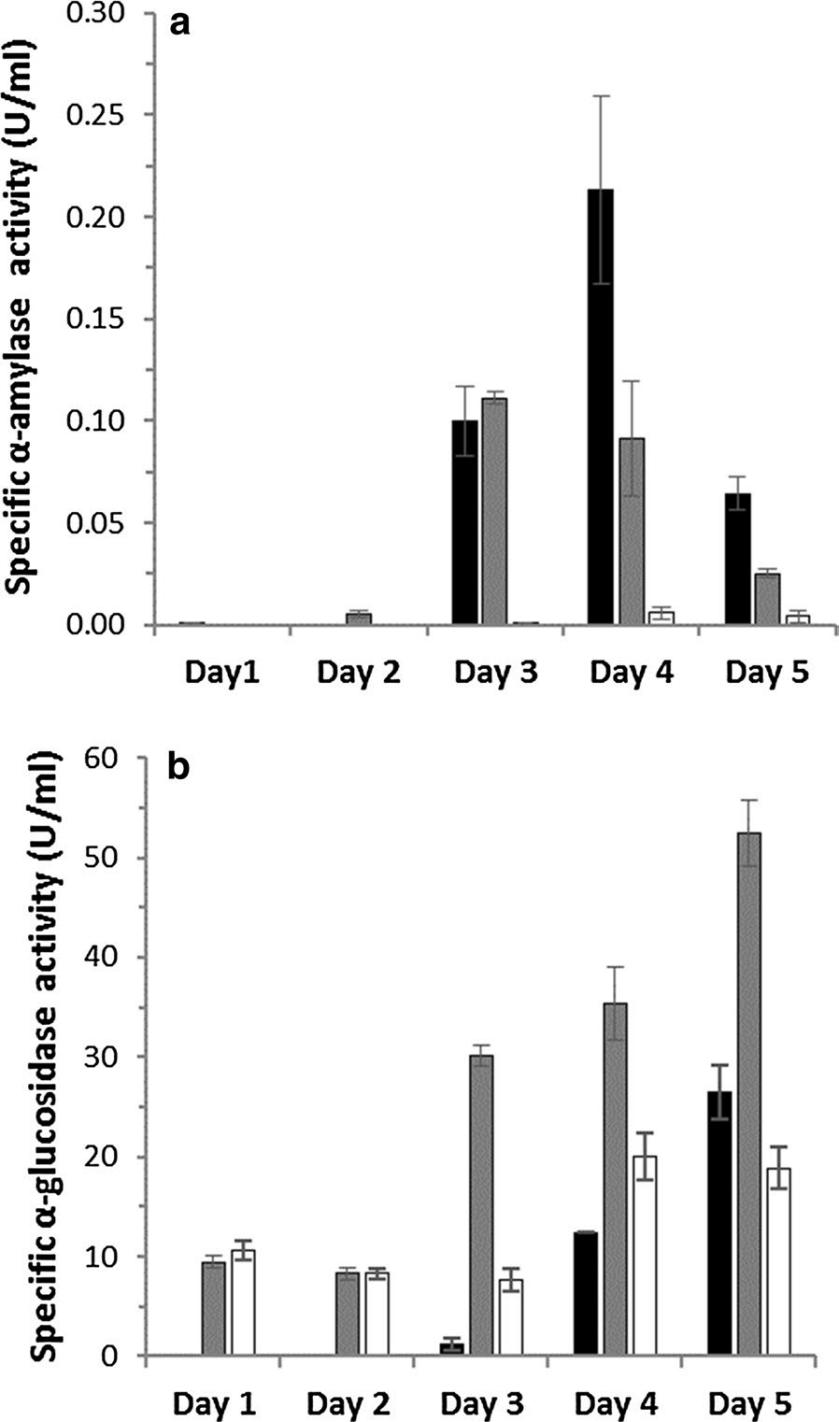
Activity of amylolytic hydrolases. Measurement of secreted α-amylase (**a**) and α-glucosidase (**b**) activities from *Aspergillus nidulans* grown on wheat (*black*), high-amylose maize (*gray*) or pea starch (*white*) for 5 days (see “Methods” section). The data are presented as means ± standard deviations of three biological replicates

The α-amylase and α-glucosidase activities were measured in the filtered culture supernatants. The average activities of the biological triplicates in different starch media at days 1–5 are shown in Fig. 1. Enzymatic activities were growth-substrate dependent and the highest α-amylase and α-glucosidase activities were measured in the wheat and maize starch culture supernatants, respectively. The α-amylase activity increased to a maximum in 3–4 days and decreased thereafter, with activity maximum (0.21 U/ml) after 4 days in wheat starch (Fig. 1a). By contrast, the α-amylase activity in the pea starch culture supernatants was barely detectable, consistent with the poor growth on this substrate.

The α-glucosidase activity increased over time in all samples and showed the highest activity (53 U/ml) in HA maize starch medium on day 5 (Fig. 1b). The glucosidase activity was roughly similar between days 1 and 3 in the pea starch culture, whereas a one-fold increase was observed at days 4–5. By contrast, the α-glucosidase activities in the HA maize and wheat cultures increased steadily until day 5 (Fig. 1b).

### Survey of secreted *A. nidulans* proteins

**Fig. 2:**
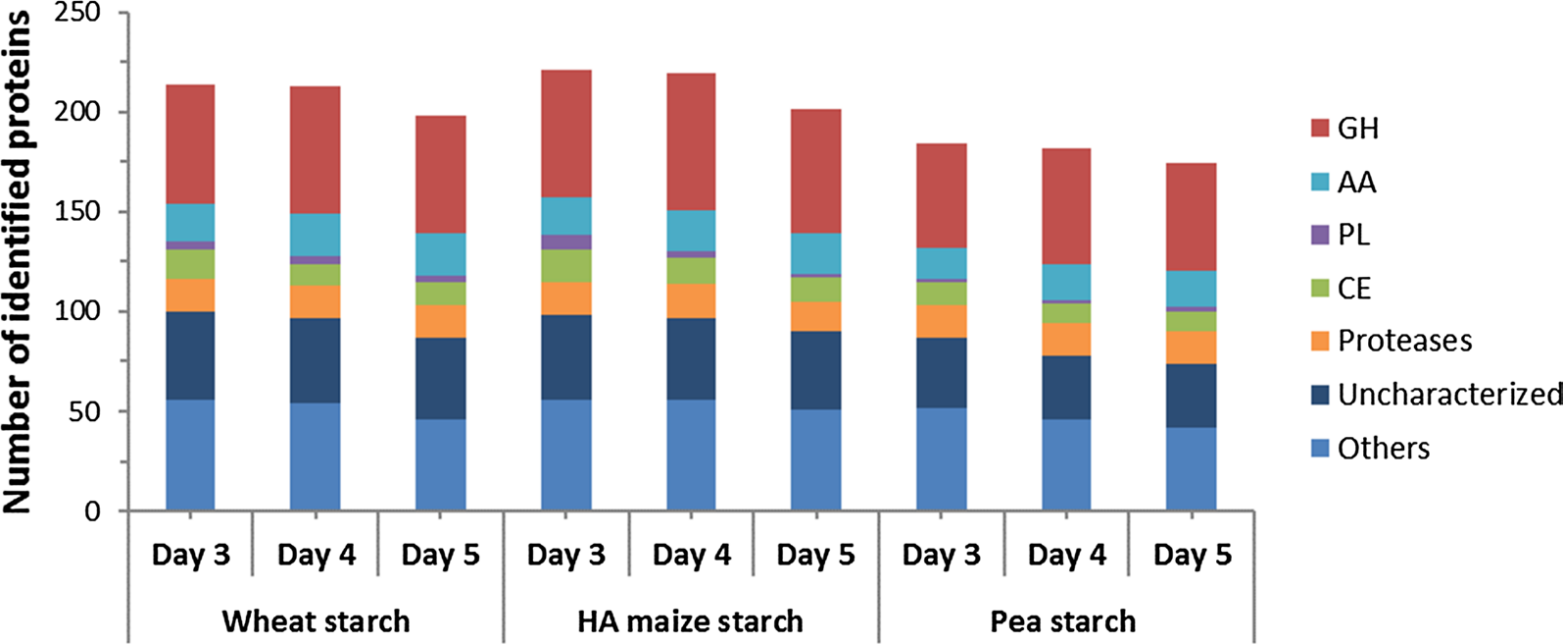
Overview of the secreted proteins in *Aspergillus nidulans* starch cultures. The figure depicts the distribution of the secreted proteins from culture supernatants of *A. nidulans* grown on wheat, high-amylose maize or pea starch on culture days 3–5. Identified carbohydrate-active enzymes (CAZymes) are assigned in the following categories: auxiliary activity (*AA*), carbohydrate esterase (*CE*), glycoside hydrolase (*GH*), polysaccharide lyase (*PL*). Secreted proteins lacking functionally characterized homologs were assigned as uncharacterized and proteases are shown to highlight their abundance. The remaining proteins belong to a variety of functional categories and are represented as “other” for clarity

**Fig. 3:**
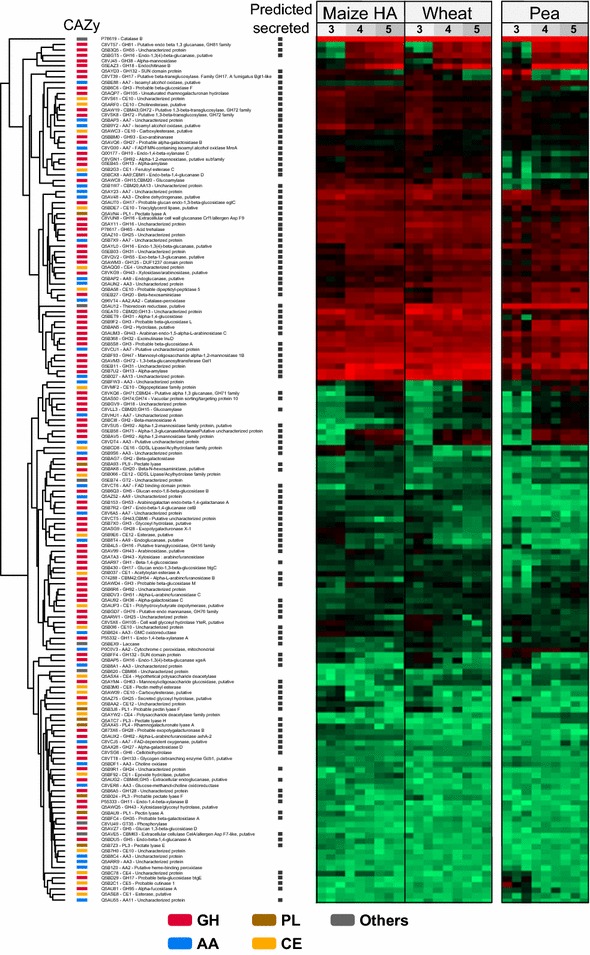
Heat map comparison of expression patterns of CAZymes detected after 3–5 days growth of *Aspergillus nidulans* on minimal media supplemented with: high-amylose (*HA*) maize, wheat, or pea starch. The colors in the heat map indicate the label-free quantification (*LFQ*) intensity reported by MaxQuant ranging from 2 × 10^6^ (*light green*) to 4 × 10^11^ (*light red*). Missing values were imputed from a normal distribution located at the quantification limit. Glycoside hydrolases (GHs) are in *red*, auxiliary activities (*AAs*) in *blue*, polysaccharide lyases (*PLs*) in *light brown*, carbohydrate esterases (*CEs*) in *yellow*, and other proteins in *gray*. Secretion prediction is a combination of SignalP, Phobius, and WolfPSort where at least two algorithms had to agree

Filtered supernatants from *A. nidulans* cultures grown on wheat, HA maize, and pea starch media were analyzed using liquid chromatography combined with tandem mass spectrometry (LC-MS/MS). The analysis of the data set (Additional file 1: Table S1) revealed dynamic secreted protein profiles over the course of 5 days. The theoretical complete proteome of *A. nidulans* contains 10,556 sequences of which 9.7 % are predicted to be secreted using a combination of three different algorithms. Of the 937 identified proteins in this study, 33 % were predicted to be secreted, which approximately represents 30 % of the theoretical secretome. The identified secreted proteins on days 3, 4, and 5 were assigned to different functional categories including proteases and various carbohydrate-active proteins and clustered both according to abundance and trend related to increase/decrease over time (Additional file 2: Figure S1, Additional file 3: Figure S2, respectively). The number of secreted proteins detected in each culture supernatant varied between 174 (pea starch, day 5) and 221 (HA maize starch, day 1) and generally the number of identified proteins decreased at day 5, as compared to days 3 and 4, but less so in pea (≈4.4 %) followed by wheat (≈7.5 %) and maize (≈9 %) starches (Fig. 2). Approximately 20 % of the secreted proteins were assigned as uncharacterized (lacking characterized homologues). For the remaining secretome, CAZymes (carbohydrate-active enzymes and proteins assigned into the CAZy database, http://www.cazy.org) represented the largest category (Fig. 3) amounting to 44 % of the secretome (Fig. 2).

Approximately 30 % of the 478 annotated CAZymes were identified in the secretomes despite growth on purified starches and not crude plant material. The data revealed that most of the identified CAZymes are GHs or AAs. Generally, the total number of identified CAZymes was lower in the pea starch cultures (Fig. 2). *Aspergillus nidulans* secreted multiple enzymes (4–10 enzymes) from certain CAZy families, e.g., GH3, GH16 and GH43, and CE10 as well as AA3, AA7, and AA9, with the three latter families harboring oxidative activities (Additional file 4: Table S2). Some ancillary carbohydrate module (CBM) families were identified including four CBM20s which mediate starch-binding in addition to cellulose-specific (CBM1 and CBM6), different β-glucans (CBM6, CBM24, and CBM43), chitin (CBM18), and arabinofuranosyl (CBM42)-binding modules.

Cell wall-degrading activities from GH5, GH6, GH7, GH53, and GH62 were present only in wheat and HA maize cultures. Beyond this, only a few families were not identified in all samples, including GH35 which was only observed in HA maize and pea starches, and GH24 and GH95 only identified in HA maize and pea starch-induced samples, respectively (Additional file 4: Table S2). Notably, a GH13 α-amylase (AmyF, Q5B7U2), a GH31 α-glucosidase (AgdB, G5EB11), and an AA13 LPMO (*An*LPMO13B, Q5B027) were the most abundant CAZymes in all samples (Fig. 3). The analysis revealed the secretion of six different putative LPMOs belonging to families AA9 and AA13 in all starch samples, and one AA11, which was only found in the pea starch culture on day 3 (Table 1; Additional file 4: Table S2).

**Table 1:** Predicted secreted lytic polysaccharide monooxygenases encoded in the *Aspergillus nidulans* genome

Protein name^a^	CBM	Uniprot	Identified^b^	Closest structural homologue^a^ (PDB ID; % sequence identity)
*An*LPMO9A		Q5BAP2	Y	*Nc*LPMO-2 (4EIR; 35)
*An*LPMO9B	CBM1	Q5BCX8	Y	*Ls*LPMOA (2AFC; 52)
*An*LPMO9C		Q5AZ52	Y	*Pc*LPMO10E (4B5Q; 49)
*An*LPMO9D		Q5B8T4	Y	*Nc*LPMO9C (4D7U; 29)
*An*LPMO9E	CBM1	Q5AQA6	N	*Ta*LPMO10A (2YET; 61)
*An*LPMO9F		Q5B6H0	N	*Ta*LPMO10A (2YET; 55)
*An*LPMO9G		Q5BEI9	N	*Ta*LPMO10A (2YET; 67)
*An*LPMO9H		Q5B7G9	N	*Ta*LPMO10A (2YET; 57)
*An*LPMO9I		Q5AUY9	N	*Ta*LPMO10A (2YET; 64)
*An*LPMO11A		Q5AU55	Y	*Ao*LPMO11 (4MAH; 51)
*An*LPMO11B		Q5BFS8	N	*Ao*LPMO (4MAH; 40)
*An*LPMO13A^c^	CBM20	Q5B1W7	Y	*Ao*LPMO13 (4OPB; 70)
*An*LPMO13B		Q5B027	Y	*Ao*LPMO13 (4OPB; 70)

^a^The putative and characterized LPMOs are designated by their names indicating the auxiliary activity (AA) family affiliation

^b^ *Aspergillus nidulans* encoded LPMOs that are identified (Y) and not identified (N) in the secretomes

^c^This enzyme has been recently shown to be a starch-active LPMO, see ref [17]

### Starch-degrading enzymes

**Fig. 4:**
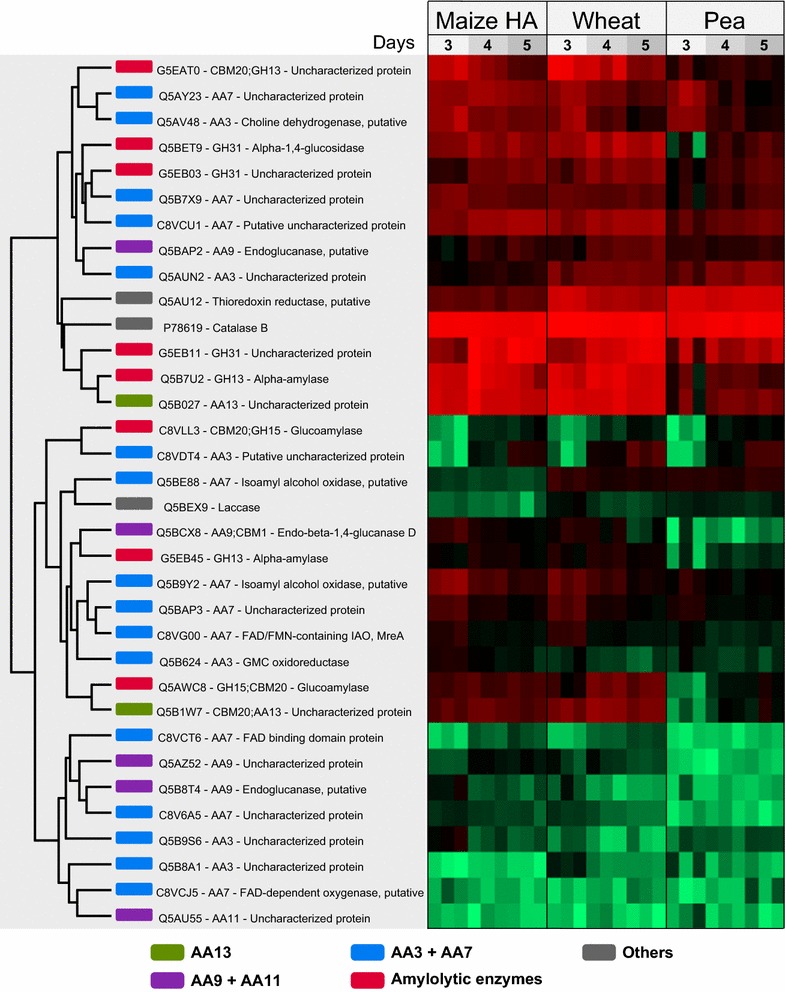
Heat map identical to Fig. 3, but filtered to contain amylolytic hydrolases and AA enzymes detected after 3–5 days growth of *Aspergillus nidulans* on minimal media supplemented with: high-amylose (*HA*) maize, wheat, or pea starch. Highlighted in *green* are AA13s, in *purple*—AA9s and AA11s, in *blue*—AA3s and AA7s, in *red*—hydrolytic enzymes associated with starch degradation, and in *gray*—other proteins

α-Amylases of GH13, glucoamylases of GH15, and α-glucosidases of GH31 are candidates for starch degradation. Indeed, seven of the twelve predicted extracellular enzymes of these families were identified in the secretome (Table 2; Fig. 4). Three putative GH13 α-amylases (G5EB45, G5EAT0, and Q5B7U2), and three GH31 α-glucosidases (G5EB03, G5EB11, and Q5BET9) were present in all samples on all days. A GH15 α-glucoamylase (C8VLL3) was identified in all wheat and HA maize starch medium and only on day 4 and 5 in pea. A second glucoamylase was also identified (GlaB, Q5AWC8) (Table 2) and included based on a previous study showing its purification from *A. oryzae* cultures [23], despite its lack of a canonical secretion signal. Moreover, the recently functionally characterized starch-degrading AA13 LPMO [17] was identified in addition to a second AA13 enzyme (Table 1; Fig. 4). Interestingly, the abundance of the GH13 α-amylase is having a CBM20 (G5EAT0) decreases over time (Fig. 4; Additional file 3: Figure S2). Otherwise, all amylolytic proteins were highly abundant at all time points or showed an increase over time (Fig. 4; Additional file 3: Figure S2).

**Table 2:** Predicted secreted enzymes assigned in CAZy families implicated in starch degradation by the *Aspergillus nidulans* genome

Protein (name)	CBM	CAZyme family	Uniprot	Identified^a^
α-amylase (AmyA)		GH13_1	G5EB45	Y
α-amylase (AmyB)	CBM20	GH13_1	G5EAT0	Y
α-amylase (AmyD)		GH13_1	Q5B822	N
α-amylase (AmyE)		GH13_1	Q5AZF6	N
α-amylase (AmyF)		GH13_1	Q5B7U2	Y
α-glucoamylase (GlaA)	CBM20	GH15	C8VLL3	Y
α-glucoamylase (GlaB)^b^	CBM20	GH15	Q5AWC8	Y
α-glucosidase (AgdA)		GH31	G5EB03	Y
α-glucosidase (AgdB)		GH31	G5EB11	Y
α-glucosidase (AgdC)		GH31	Q5AWI5	N
α-glucosidase (AgdE)		GH31	Q5BET9	Y
α-glucosidase		GH31	Q5AU13	N

SignalP was used to predict secretion signals

*GH* Glycoside hydrolase

^a^Putative starch-degrading proteins identified (Y) and not identified (N) in the *A. nidulans* secretome

^b^ This enzyme lacks a canonical secretion signal, but was included based on [23]

### Redox-active enzymes

The *A. nidulans* genome encodes 13 LPMOs: 9 AA9s, 2 AA11s, and 2 AA13s (Table 1). Two AA9 are assigned in clade LPMO1, one in LPMO2, five in clade LPMO3, and one in an unclassified cluster, as phylogenetically defined by Vu et al. [8]. None of the five clade 3 LPMOs were identified, whereas the remaining four enzymes were observed, albeit at varying abundance (Table 1; Fig. 4). The two AA13 LPMOs were among the most abundant proteins in the culture supernatants, *An*LPMO13B (Q5B027) seemingly at higher abundance than *An*LPMO13A (Q5B1W7). The N-terminal histidine residue of both these AA13 enzymes seemed to be methylated in virtually all samples (only exception being the day 2,3 replicates from the pea culture) based on the identification of the “H(Me)GYLTVPASR” peptide (best MASCOT score = 78) that is identical in both enzymes, while the non-methylated counterpart could not be detected. Notably, the abundance of both proteins increased slightly over time (Fig. 4; Additional file 3: Figure S2).

In addition to LPMOs, the secretomes also contain a variety of other redox-active enzymes, including a catalase, laccase, and thioredoxin reductase as well as members of AA3 and AA7 (Fig. 4; Additional file 3: Figure S2). The six (out of 18) putative AA3 enzymes secreted by *A. nidulans* belong to sub group 2 that accommodates aryl-alcohol and glucose oxidases as well as dehydrogenases [24]. Of these, two (Q5AV48 and Q5AUN2) were highly abundant, whereas the initially low abundance C8VDT4 shows a substantial increase over time (Fig. 4; Additional file 3: Figure S2). The remaining AA3s are only present at low abundance. *A. nidulans* secretomes show the presence of 10 AA7 putative glyco-oligosaccharide oxidases (GOOs), three of which (Q5AY23, Q5B7X9, and C8VCU1) were highly abundant in most samples, the two former and the latter showing a decreasing and increasing trend over time, respectively (Fig. 4; Additional file 3: Figure S2).

The secretomes contain several non-CAZyme redox-active enzymes. As a matter of fact, the most abundant protein in all collected supernatants (except day 5 maize and day 3 pea) is Catalase B (CatB, P78619), which also shows a slight decrease in abundance over time in wheat and maize, and an opposite trend in pea (Fig. 4; Additional file 5: Tables S3–S5). Thioredoxin reductase is abundant in most samples (among top 20 proteins in wheat and pea starch samples, Additional file 5: Tables S3 and S5) and shows a decreasing trend in the wheat and maize samples like CatB. A laccase (Q5BEX9) is also present in the secretome at low abundance, and more so over time.

### Plant cell wall-degrading non-oxidative enzymes

Ten putative β-glucoside and cellulose-degrading enzymes were identified. A GH1 (Q5AR97) and five putative GH3 β-glucosidases (Q5B5S8, Q5B6C6, Q5AWD4, Q5B7X0, and Q5B9F2) were identified in all samples. Two of these enzymes (Q5AR97, Q5B7X0) appeared only after 4 days in the pea starch. Notably, cellobiohydrolases (GH6) and endoglucanases (GH5_7) active on cellulose were not identified in the pea starch culture supernatant. Two GH5 (Q5AUG2, Q5BDU5) were only present in the maize, whereas the GH6 (C8VSG6) and GH7 (Q5B7R2) were also identified in wheat starch cultures. Twelve putative hemicellulose-degrading enzymes were identified including a GH10 (Q00177) and a GH11 (P55332) β-1,4-endoxylanases and four putative GH43 β-xylosidases/α-l-arabinofuranosidases (C8VCT5, C8VKG9, Q5AUM3, and Q5AV99). α-Arabinofuranosidases of GH54 (O74288) and GH62 (Q5AUX2) were also identified, the latter only in wheat and HA maize starch media together with a GH53 β-1,4-endogalactanase (Q5B153). Moreover, a putative GH93 exo-arabinanase (Q5BBM0), a GH27 (Q5AX28) and a GH36 (Q5AU92), both the latter being α-1,6-galactosidases were identified. Putative pectin-degrading enzymes including a GH2 β-glucuronidase (Q5BAN5), a GH105 unsaturated rhamnogalacturonan hydrolase (Q5AQP7), and two putative GH28 exo-polygalacturonases (Q5ASG9 and Q873X6) were detected, the latter only in HA maize starch cultures. Finally, a GH35 putative β-galactosidase (Q5BFC4) was detected in HA maize and pea starch cultures.

### Fungal cell wall active enzymes

Fifteen secreted enzymes putatively involved in fungal cell wall modification and degradation were identified including six GH16 glycanases (C8VUN8, Q5AY11, Q5AYL0, Q5BAP5, Q5BGT5, and Q5B4L5), a GH17 β-1,3-endoglucanase (Q5AUT0), two putative GH55 β-1,3-exoglucanase (Q5B3Q5 and C8VQV2), three GH72 β-1,3-glucanosyltransferases (C8VSK8, Q5AVM3, and Q5AW19), a GH81 endo-β-1,3-endoglucanase (C8VT57), and a putative GH20 *N*-acetylglucosaminidase (G5EB27). The putative GH71 α-1,3-glucanase (C8VKQ6) was detected only on days 4 and 5 in all cultures. In addition, three α-mannan-degrading enzymes, one GH47 (Q5BF93), and two GH92 (C8VSU5 and Q5BAV5) were identified.

### Purification of starch-binding enzymes from wheat starch cultures

**Fig. 5:**
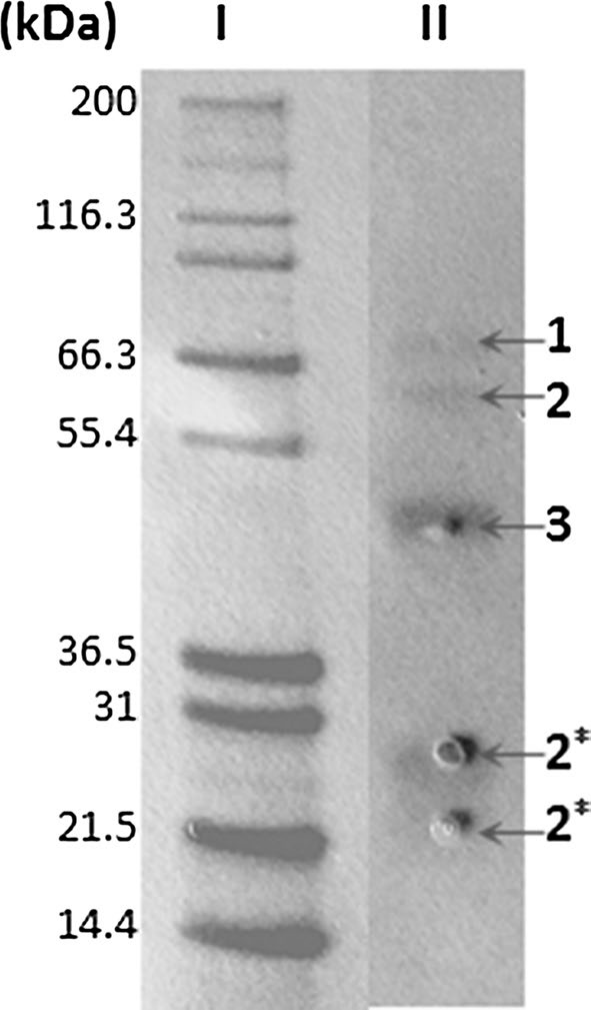
Purification of starch-binding enzymes from the secretome. β-cyclodextrin-sepharose affinity chromatography purification of the *Aspergillus nidulans* wheat starch culture supernatant harvested after 5 days (*lane II*). Three dominant bands were identified using mass spectrometry (MALDI-TOF MS, see “Methods” section). The bands were identified as glucoamylase (*1*), α-amylase (*2* and 2*-CBM20 domain), and *An*LPMO13A (*3*). Molecular mass marker in kDa (*lane I*)

β-Cyclodextrin affinity chromatography was used to capture the fraction of *A. nidulans* secreted enzymes that possess affinity for starch. The elution fraction from this purification was analyzed by SDS-PAGE (Fig. 5) and dominant protein bands were identified using MALDI-TOF mass spectrometry (Table 3). The common feature to all proteins identified on the gel is the presence of CBM20 [25]. The three most intense bands contain the GH15 α-glucoamylase (GlaB, Q5AWC8), the GH13_1 α-amylase (G5EAT0), and the biochemically characterized starch-active LPMO *An*AA13A (Q5B1W7, Table 1), that appeared as the most abundant protein in this starch-associated secretome fraction. The CBM20 of the α-amylase was also identified in two lower molecular mass bands on the gel, which is likely due to proteolytic cleavage (Fig. 5).

**Table 3:** Identification of β-cyclodextrin-sepharose affinity chromatography purified secreted proteins in *Aspergillus nidulans* culture grown on wheat starch for 5 days

Protein/module	Protein family	Uniprot	MW^a^ (kDa)	*p*I^a^	Score^b^	Sequence coverage^b^, %
LPMO/CBM20	AA13	Q5B1W7^c^	42	4.7	138	16
α-amylase/CBM20	GH13	G5EAT0	69	4.8	85	6
α-glucoamylase/CBM20	GH15	Q5AWC8	71	5.3	211	6

^a^Hypothetical molecular weight and *p*I of the proteins

^b^The score and sequence coverage (%) are based on the genome sequence information. MASCOT scores >26 indicate identity or significance threshold (*p* < 0.05)

^c^This LPMO has been recently shown to be active on starch [17]

## Discussion

The abundance of starch from terrestrial biomass is only surpassed by cellulose. An excess of two billion tons of starch are annually produced from cereals and coarse grains and another 700 million tons from roots and tubers (The food and agriculture organization of the United Nations [FAO]; http://www.fao.org/). The bulk of extracted starch from these crops is used for non-food industrial applications including first generation biofuel that still contributes more than two thirds of the total biofuel production [26]. Technical and economic issues continue to hamper a larger transition to second generation lignocellulose-based feedstocks [27], which together with the large investments made in first generation production facilities is likely to maintain the demand for starch as a feedstock in the time to come. Therefore, improving the efficiency of starch depolymerization in industrial applications has significant economic and environmental potential.

The synergistic action of LPMOs with hydrolases [10, 28] in the breakdown of recalcitrant polysaccharides has spurred interest in the exploitation of these enzymes in biofuel production [11]. Several proteomics studies revealed that LPMOs are highly co-secreted with hydrolases during fungal growth on plant-derived biomass or purified polysaccharides [20, 29]. The oxidative activity of starch-specific LPMOs from *Neurospora crassa* [16] and *A. nidulans* [17] toward resistant retrograded starch was recently demonstrated and the presence of the *A. nidulans* LPMO elicited a 100-fold increase in the release of maltose by a commercial β-amylase on the same substrate [17]. This in vitro activity suggests that starch-active AA13 LPMOs may play an important role in starch depolymerization by distinct fungi. Currently, there are no data on the involvement and biological significance of LPMOs in starch degradation. In this study we investigate the protein inventory secreted by *A. nidulans* during growth on three different starches differing in botanical origin and properties and show that starch-specific LPMOs are highly abundant in all starch secretomes together with LPMOs of different specificities and other oxidative enzymes.

### *A. nidulans* secretomes are fine-tuned to the different starch matrices

Fungal growth was far better on cereal starches from wheat and maize, while the pea starch was a poor growth substrate. The overall secretion profiles differed between the three substrates, but were more similar between the botanically closer cereal starches as compared to pea (Fig. 3; Additional file 2: Figure S1). This was also supported by enzyme assays that revealed much higher α-glucosidase and α-amylase activities in the cereal starch cultures and a substantial increase of α-amylase activity at day 3, which was not observed in the pea starch culture (Fig. 1). This is also consistent with large differences in the 20 most abundant proteins (Additional file 5: Tables S3–S5). The diversity of identified CAZymes was the highest in HA maize starch and the lowest in pea starch secretomes that contained a larger relative proportion of unknown enzymes (Fig. 2). These data highlight the fine-tuning of the secretomes to each starch substrate. The three used starches are different in their fine structures and composition. The HA maize starch has the highest amylose content with approximately 70 % (w/w), followed by pea starch (>45 % w/w) and wheat (25 % w/w). HA maize starch granules are the smallest (5–25 µm), followed by pea (5–45 µm) and wheat (20–35 µm) [3, 4]. Given the chemical simplicity of starch, it is unlikely that the secretomes merely reflect the differences in starch structure per se, but also the presence of other non-starch components, including polysaccharides, lipids, and proteins, which may impede the action of amylolytic enzymes [30]. The starch granules and protein storage vacuoles in monocot cereals like wheat and maize reside in the endosperm, while in pea the cotyledon tissue serves the same storage function [1, 31]. Pea starch isolation has been reported to be more difficult due to the presence of fine fiber and insoluble flocculent proteins, significant amounts of which remain associated with the starch [32]. Indeed, numerous proteases are identified in the secretomes and more so in the pea than the cereal starch cultures (Fig. 2; Additional file 2: Figure S1). Pea starch is generally less well studied with respect to microbial degradation and the reasons for the poorer growth on this substrate are unclear. Starch from HA maize has been shown to be the least efficiently degraded by an α-amylase from *Aspergillus fumigatus*, followed by starch from pea [33]. The excellent growth of *A. nidulans* on the more resistant HA maize starch supports the possibility that non-starch components in the pea starch matrix rather than the recalcitrance of the starch is responsible for the poor growth. Indeed, legume cotyledons including pea are known to harbor a range of protease and hydrolase inhibitors [34], which may represent more challenging growth conditions and delay the deconstruction of the starch.

The major components in the cereal endosperm cell walls are arabinoxylans and mixed linkage glucans, in addition to small amounts of cellulose, xyloglucan, and pectins [35]. By contrast, xyloglucan is the main structural polysaccharide in cotyledon cell walls [36]. This is consistent with the presence of cellulases of GH5, GH6, and GH7, in addition to GH62 arabinoxylan arabinofuranosidase exclusively in the cereal starch cultures (Fig. 3; Additional file 4: Table S2). Xylose has been suggested to be the inducer of several polysaccharide-degrading enzymes in aspergilli [37]. Possibly the co-secretion of a range of hemicellulose-degrading enzymes including a GH10 xylanase with amylolytic enzymes (Fig. 3; Additional file 3: Figure S2) is suggestive of the presence of xylose derived either from arabinoxylan in cereals or xyloglucan remaining in the legume pea starch preparations. This co-secretion may contribute to the degradation of cell wall components and render starch more accessible to the action of amylolytic enzymes. An “anti-caging” effect of xylanases and other cell wall hydrolases has been observed in vitro starch digestion studies [38, 39]. Also the secretion of five LPMOs of AA9, which feature in the breakdown of cellulose and hemicellulose (including an enzyme with a putative cellulose binding module, Table 1) supports the presence of cell wall components in the starch matrices [7, 13, 14]. These findings motivate further studies on the effect of low concentrations of key hemicellulose polysaccharide-degrading enzymes on the yields of starch degradation.

### The hydrolytic starch-degrading machinery

The genomic and enzymatic capabilities of *A. nidulans* with regard to complex polysaccharide degradation [19, 40, 41] manifest the saprophytic lifestyle of this fungus. The starch degradation model in fungi has been based on the biochemically well-described amylolytic hydrolases from aspergilli [33, 42]. The present study is the first to report high resolution secretome data in response to growth on different starches, providing insight on the impact of starch botanical origin on growth and protein secretion profiles.

The data revealed the secretion of a core of starch hydrolytic battery of *A. nidulans* comprising three α-amylases, three α-glucosidases, and two glucoamylases (Table 2). The absence of AmyD and AmyE of GH13_1 from the secretome (Table 2) is in excellent agreement with the fungal cell wall remodeling role of these enzymes [43]. An intriguing observation is the decrease in abundance of the GH13-CBM20 (AmyB, G5EAT0) over time (Fig. 4; Additional file 3: Figure S2). The capture of the CBM20 proteolytic degradation product of this enzyme from the starch-binding fraction of the secretome (Fig. 5) proves proteolytic degradation has occurred, which is also in agreement with the decrease of α-amylase activity on day 5 in cereal starches, despite the slight increase in abundance of the other α-amylase (AmyF) not possessing a CBM20. By contrast, a pattern of increased abundance is observed for the CBM20-containing LPMO (*An*LPMO13A, Q5B1W7) and GH15 glucoamylases (Fig. 4; Additional file 3: Figure S2). This expression pattern suggests that the starch-binding α-amylase AmyB (G5EAT0) plays an instrumental role in the initial attack on granular starch. It is tempting to speculate that the endo-acting activity of AmyB creates sites for attack by both glucoamylases and α-glucosidases that release glucose from the non-reducing ends of starch and solubilized fragments thereof. The high abundance and relative increase in AA13 LPMOs with time, observed here for the first time, indicate that these activities are of great importance in starch deconstruction. The possible increase of resistant starch structures that escape hydrolytic degradations during growth may justify maintained or increased levels of these enzymes that have been shown to boost the degradation of resistant starch, toward the later stages of growth, which will be further discussed below.

### Abundant deployment of LPMOs in starch degradation by *A. nidulans*

Several studies investigating the secretomes of plant biomass-degrading microorganisms have reported the abundant presence of LPMOs, indicating their importance in biomass conversion [20, 29]. However, the significance of LPMOs in starch utilization in vivo has not been addressed. Two studies recently reported oxidative cleavage of starch by fungal AA13 LPMOs [16, 17]. Indeed, LPMOs from both family AA9 and AA13 were identified in the starch secretomes, the latter family being the most abundant. As a matter of fact, one of the AA13 LPMOs, *An*LPMO13B (Q5B027) is among the top four most abundant proteins at all time points in the wheat and maize starch cultures (Fig. 4; Additional file 5: Tables S3, S4), indicating a prominent role in starch degradation. The other AA13 LPMO (*An*LPMO13A), which has a CBM20 appended, is also abundant in the secretomes of cereal starches (always among the top 10 %). The abundance of *An*LPMO13A may be underestimated as compared to *An*LPMO13B, due to the preferential CBM20-mediated binding of the former onto granular starch (concluded form β-cyclodextrin capture, Fig. 5). *An*LPMO13A has already been demonstrated to cleave amylose chains and to boost hydrolysis of retrograded starch [17], whereas no activity data are available for *An*LPMO13B. However, the two AA13 LPMOs are highly similar (catalytic modules share 81 % sequence identity), indicating that they also share substrate specificity. A notable difference is that *An*LPMO13B possesses a 75 amino acid C-terminal extension of unknown function as compared to the long linker and the C-terminal CBM20 of *An*LPMO13A. The absence of *An*LPMO13B in secretome fraction captured by β-cyclodextrin affinity suggests that the affinity of this enzyme to insoluble starch is much lower than *An*LPMO13A that possesses a CBM20. These differences indicate that the AA13 LPMOs may target different parts of the starch structure, yielding complementary activities. This has been observed for the conserved cellulose-targeting LPMO pair encoded by *Streptomyces coelicolor*, which contributed synergistically when combined in cellulose degradation [44]. Interestingly no activity of the AA13 catalytic module lacking the CBM20 was detected [17]. This highlights the role of the CBM20 in targeting the enzyme to the insoluble starch structures.

Although less abundant than the family AA13 LPMOs, four AA9 LPMOs and one AA11 LPMOs were identified in the cultures (Table 1; Additional file 4: Table S2). So far, AA9 LPMOs have only been shown to depolymerize cellulose and hemicellulose substrates. Thus, it is reasonable to speculate that these enzymes are secreted by the fungus to aid gaining access to the starch granules that in vivo are shielded by a protective lignocellulose layer, e.g., grain bran or to disrupt cell walls in the starchy endosperm tissue. Interestingly, the five clade 3 AA9 LPMOs of the *A. nidulans* genome were not represented in the secretomes (Table 1). Clade 3 LPMOs are proposed to have mixed activity, yielding both C1- and C4-oxidized products, whereas clade 1 members oxidize the C1 carbon and clade 2 the C4 carbon of the substrate. The significance of this expression pattern is not known, but may be related to the type of substrate the fungus is exposed to.

One of the two AA11 LPMOs is observed in the secretomes, albeit at low abundance. The enzyme shows only moderate similarity to the chitin-degrading *Ao*AA11 (51 %) [45], precluding reliable functional assignment. Assuming chitin specificity (like *Ao*AA11), it is possible that the function is related to fungal cell wall remodeling.

### The role of non-LPMO redox-active enzymes in starch degradation

It is well established that the sub-family 1 members of the glucose, methanol, choline (GMC) oxidoreductases (AA3s), i.e., the cellobiose dehydrogenases (CDHs), can provide electrons to LPMOs [28, 46]. Maltodextrins are known as poor substrates for CDHs [47], thus it may be that other redox-active enzymes play this role during starch degradation. Indeed, multiple enzymes from the AA3 family and the AA7 family (glyco-oligosaccharide oxidases with one enzyme shown to be active on maltose other malto-oligosaccharides) are identified in the culture supernatant (Fig. 4).

Both aryl-alcohol oxidases and glucose dehydrogenases, both assigned into AA3_2, have been, very recently, shown to be able to transfer electrons to LPMOs [24, 48]. Two (C8VDT4 and Q5B9S6) of the six identified putative AA3_2 enzymes in the present study are not currently assigned in CAZy, whereas the remaining four sequences populate clusters lacking any biochemically characterized enzymes in a recent phylogenetic analysis of AA3_2 [49], which precludes a reliable assignment of their activity. Notably, the two identified AA3_2 putative enzymes Q5AUN2 and Q5B624 populate a single cluster adjacent to characterized glucose dehydrogenases in the same phylogenetic analysis [49]. The identified AA7 enzymes (and possibly some of the AA3_2) are predicted oxidases (not dehydrogenases as the CDHs), indicating that one of their main products is H_2_O_2_. A possible role for enzymes generating H_2_O_2_ is to provide lignin-degrading enzymes like laccases and peroxidases with hydrogen peroxide, required for lignin depolymerization. On the other hand, no or little lignin is present in the substrates provided in the present experiments, possibly indicating a different role of these enzymes. Biochemical characterization of the identified AA7 and AA3 enzymes is warranted to verify their roles.

The H_2_O_2_ generated from the activity of AA3 and AA7 oxidases on products derived from depolymerization of starch (or other components in the substrate matrices) provides a rationale for the dominance of catalase B (CatB) in most secretomes. Catalase catalyzes the conversion of H_2_O_2_ to O_2_ and H_2_O, thus protecting the fungus from H_2_O_2_ toxic effects (i.e., hydroxyl radicals emerging from Fenton chemistry). Indeed, the expression of CatB has previously been demonstrated to be induced by H_2_O_2_ or H_2_O_2_-generating conditions [50], and the enzyme activity was suggested to protect the fungus from the toxic side-products encountered in aerobic growth. It should be noted that LPMOs, which represent some of the most abundant proteins in the cultures, are capable of substantial H_2_O_2_ production [51]. A second highly abundant protein observed in the fungal cultures is thioredoxin reductase (Fig. 4; Additional file 5: Tables S3–S5), a protein also related to detoxification of reactive oxygen species (ROS) [52]. Thus, it seems that the fungus is actively protecting itself from ROS during aerobic degradation of biomass. These findings could inspire future research on design of enzyme cocktails for biomass depolymerization, i.e., protecting enzymes from toxic byproducts formed by oxidative enzymes. As a matter of fact, one study has already shown the advantageous use of catalase in enzyme cocktails containing high levels of LPMOs [53].

### AmyR regulation of amylolytic hydrolases and LPMOs

The AmyR transcription factor, that is activated by binding isomaltose, has been shown to regulate the expression of the α-glucosidases agdA, agdB, and agdE agdF, the α-amylases *amyA*, *amyB*, and *amyF*, and the glucoamylase glaB genes in *A. nidulans* [54]. All these enzymes were indeed identified in the present study (Table 2). Analysis of the *A. nidulans* genome revealed the presence of the AmyR consensus sequence 5′-CGGN_8_CGG-3′ at around −300 basepairs (−318 to −305) in the promotor region of the *An*LPMO13A and around −600 basepairs (−612 to −609) for *An*LPMO13B. This is the first time a common regulatory link has been identified between GHs and LPMOs. Interestingly, hierarchical clustering analysis revealed that both AA13 enzymes have very similar secretion patterns as distinct amylolytic enzymes under the control of AmyR (Fig. 4). These first data on the temporal distribution of starch-degrading enzymes suggest a sophisticated regulatory mechanism, whereby the fungus deploys specific enzymes at different stages of the starch growth. As mentioned above, it is tempting to speculate that the increased secretion of starch-specific AA13s correlates with the later stages of the culture, where the more resistant structures in the starch substrate accumulate. Further studies are needed to unveil the precise preference of the two types of AA13 enzymes observed in this study.

## Conclusions

This is the first study that uses high resolution secretomics analysis to probe the temporal changes of protein profiles of the model saprophytic ascomycete *A. nidulans* growing on different starchy substrates. The data, for the first time, unveil a conspicuous abundance of AA13 LPMOs in the secretomes, which is suggestive of an instrumental role of these enzymes in starch degradation. Another novel finding is the identification of binding sites of the transcriptional regulator AmyR, which established a co-regulatory link between GHs and LPMOs featuring in starch degradation. Beyond a core amylolytic machinery, the secretomes were clearly correlated to the starch type used for growth and numerous CAZymes, proteases, and oxidoreductases were secreted. A possible rationale for this is targeting non-starch component in the substrate matrix. The abundance and co-secretion patterns of LPMOs, AA3, AA7, and other oxidative enzymes is particularly intriguing and merits further work to understand the role of these enzymes. This novel insight promotes our understanding of the degradation of different starches, including those of more resistant nature, and inspires formulation of better commercial enzyme cocktails for more efficient exploration of this important biomass resource.

## Methods

### Carbohydrate substrates and assay chemicals

The wheat starch was from Sigma-Aldrich (S5127, unmodified), pea and high-amylose (HA) maize starches were from KMC (Brande, Denmark). The starches for growth experiments were not autoclaved to minimize changes of the native starch granule structure, but washed twice in 70 % ethanol and water and subsequently pelleted by centrifugation (14,000×*g,* 5 min) before resuspension in autoclaved growth media or as substrates for enzymatic assays. *p*-Nitrophenyl-α-d-glucopyranoside (PNPG) and *p*-nitrophenol (PNP) were from Sigma-Aldrich, and insoluble Blue Starch was a custom preparation from Pharmacia (Uppsala, Sweden).

### Fungal strain and culture conditions

*A. nidulans* strain FGSC A4, (FGSC, Kansas City, MO), was pre-grown on malt extract agar plates containing 1 % wheat, HA maize or pea starches for 5 days at 30 °C until new mycelia were formed. Mycelial plugs were used to inoculate 1 L of minimal medium containing 1 % (w/v) carbon source with a start pH of 6.5 in 3 L baffled shake flasks. The minimal medium contained, per liter, 6 g NaNO_3_, 0.52 g KCl, 0.52 g MgSO_4_·7H_2_O, 1.52 g KH_2_PO_4_, and 2 ml of Hutner’s trace elements. Hutner’s trace elements contained, per liter, 2.2 g ZnSO_4_·7H_2_O, 1.1 g H_3_BO_3_, 0.5 g MnCl_2_·4H_2_O, 0.5 g FeSO_4_·7H_2_O, 0.16 g CoCl_2_·6H_2_O, 0.16 g CuSO_4_·5H_2_O, 0.11 g (NH_4_)Mo_7_O_24_·4H_2_O, and 5 g EDTA. The cultures were incubated on a rotary shaker (150 rpm) at 30 °C for 5 days. All experiments were performed in biological triplicates. The medium was supplemented with antibiotics (100 µg/ml ampicillin and 34 µg/ml chloramphenicol) to inhibit growth of possible bacterial contamination from the starch substrates.

### α-amylase and α-glucosidase activities

Both enzymatic activities were measured and averaged from the biological triplicates.

α-amylase activity of the fungal cultures was assayed toward insoluble Blue Starch (iBS). The activity was measured using iBS (6.25 mg/ml) suspended in 10 mM MES, pH 6.5. The reaction mixture (900 µl) was incubated for 15 min at 37 °C after addition of the culture filtrate (100 µl). The reaction was stopped by addition of 0.5 M NaOH (200 µl). After centrifugation (4000×*g*, 3 min) 200 µl supernatant was transferred to a 96 well microtiter plate and *A*_620_ values were used to determine enzyme activity. One activity unit (U) was defined as the amount of enzyme that leads to an increase in *A*_620_ of 1 absorbance unit in the reaction mixture under these experimental conditions.

The α-glucosidase activity was determined using *p*-nitrophenyl α-d-glucopyranoside (PNPG) as the substrate. The activity in culture filtrates was assayed toward 2 mM PNPG in 10 mM MES, 0.005 % Triton X-100, pH 6.5 and 10 μl culture supernatant in 50 μl reactions, at 37 °C for 60 min. The reaction was stopped by adding 1 M Na_2_CO_3_ (200 μl). The amount of released *p*-nitrophenol (PNP) was measured spectrophotometrically at *A*_410_ using PNP (0–2 mM) as a standard. One unit of activity (U) was defined as the amount of enzyme that released 1 μmol/min of PNP at the given conditions.

### Preparation of secretome samples and mass spectrometry analysis

Culture filtrates containing the fungal secretomes were separated from mycelia and residual insoluble starch by centrifugation (15,000×*g*, 15 min, 4 °C) and filtration (0.45 µm hydrophilic PVDF membranes; Millipore) and stored at −20 °C until further use. Samples (10 ml) from three biological replicate cultures were collected from day 3 to 5 under sterile conditions. Protein concentrations of the culture filtrates were determined using a Bradford assay (Bio-Rad Laboratories, Hercules, CA) with a BSA standard according to the manufacturer’s instructions.

Proteins of culture filtrates (2 ml) were precipitated by direct addition of 500 µl cold 50 % trichloroacetic acid (TCA), incubated at 4 °C for 8 h, centrifuged (15,000×*g*, 15 min, 4 °C) to pellet precipitated protein and washed with 300 µl ice-cold 10 mM HCl in 90 % acetone. After centrifugation (15,000×*g*, 15 min, 4 °C), pellets were dried, redissolved in 100 µl 20 mM Tris–HCl at pH 8, reduced with 10 mM (final concentration) dithiothreitol (DTT) and incubated at room temperature for 30 min. Alkylation was performed by adding 15 mM (final concentration) iodoacetamide (IAA) and incubating at room temperature for 30 min in the dark. The proteins were digested with 20 µl 12.5 ng/µl sequencing-grade modified trypsin (Promega, Madison, WI) and incubated at 37 °C for 16 h. Trypsination was stopped with 0.5 % (final concentration) trifluoroacetic acid (TFA). Samples were dried in SpeedVac and purified with ZipTip C18 pipette tips (Merck Millipore, Cork, Ireland) according to manufacturer’s instructions. Purified samples were dried and dissolved in 2 % acetonitrile (ACN) and 0.1 % TFA mix.

The peptides were analyzed in two technical replicates using a nanoHPLC-MS/MS system consisting of a Dionex Ultimate 3000 RSLCnano (Thermo Scientific, Bremen, Germany) connected to a Q-Exactive hybrid quadrupole-orbitrap mass spectrometer (Thermo Scientific) equipped with a nano-electrospray ion source. Samples were loaded onto a trap column (Acclaim PepMap100, C_18_, 5 µm, 100 Å, 300 µm i.d. ×5 mm, Thermo Scientific) and backflushed onto a 50 cm analytical column (Acclaim PepMap RSLC C_18_, 2 µm, 100 Å, 75 µm i.d., Thermo Scientific). At the start, the columns were in 96 % solution A (0.1 % (v/v) formic acid), 4 % solution B (80 % (v/v) ACN, 0.1 % (v/v) formic acid). Peptides were eluted using a 90 min gradient from 4 to 13 % (v/v) solution B in 2 min, 13–45 % (v/v) in 70 min and finally to 55 % B in 5 min before the wash phase at 90 % B, all at a flow rate of 300 nl/min. To isolate and fragment the ten most intense peptide precursor ions at any given time throughout the chromatographic elution, the Q-Exactive mass spectrometer was operated in data-dependent mode to switch automatically between orbitrap-MS and higher-energy collisional dissociation (HCD) orbitrap-MS/MS acquisition. The selected precursor ions were then excluded for repeated fragmentation for 20 s. The resolution was set to R = 70,000 and R = 35,000 for MS and MS/MS, respectively. For optimal acquisition of MS/MS spectra, automatic gain control (AGC) target values were set to 1,000,000 charges and a maximum injection time of 128 ms.

MS raw files were imported into MaxQuant [55, 56] version 1.4.1.2 and proteins were identified and quantified using the MaxLFQ algorithm [57]. The samples were searched against a database containing the total theoretical proteome of *A. nidulans* downloaded from UniProt (10,557 sequences) [58] supplemented with common contaminants such as keratins, trypsin, and BSA. In addition, reversed sequences of all protein entries were concatenated to the database to estimate the false discovery rate (FDR). Protein N-terminal acetylation, oxidation of methionine, conversion of glutamine to pyroglutamic acid, and deamidation of asparagine and glutamine were used as variable modifications, while carbamidomethylation of cysteine residues was used as a fixed modification. Trypsin was used as digestion enzyme and two missed cleavages were allowed. The ‘match between runs’ feature of MaxQuant was enabled with default parameters, to transfer identifications between samples based on accurate mass and retention time [57]. This was done to increase the number of identified peptides and set so that transfer of peptides was only allowed between samples from the same substrate. All identifications were filtered to achieve a protein FDR of 1 % and two ratio counts were required for a valid protein quantification. To evaluate the active site N-terminal histidine residue in both AA13 enzymes methylated, we performed a separate search using Mascot [59] including a variable modification of histidine-methylation as well as semitryptic cleavage pattern to obtain N-terminal matches after signal peptide cleavage.

Post-processing was done using Perseus version 1.5.0.31. Proteins categorized as only identified by site and matches to reversed sequences or contaminants were removed. Furthermore, the proteins were filtered so that a valid quantification existed for at least two of the three replicates on at least one substrate. Intensities were log-transformed and missing values imputed based on a downshifted normal distribution. Hierarchical clustering and heat map generation were done with Euclidean distance measure and average linkage. For visualization of trending proteins, ANOVA (permutation-based FDR, p < 0.10) was used to filter out proteins that showed no significant change over time. Protein quantitative values were z-scored within substrate and imputed values removed prior to clustering and heat map generation. Secretion prediction was a combination of SignalP [60], Phobius [61] and WoLF PSort [62] where at least two prediction algorithms had to agree. Secreted CAZymes were annotated using dbCAN (http://csbl.bmb.uga.edu/dbCAN/) [63].

### β-Cyclodextrin affinity chromatography purification of secreted proteome and identification of most abundant proteins

The supernatant of the culture grown on wheat starch for 5 days was supplemented with (NH_4_)_2_SO_4_ to 0.5 M final concentration and agitated until the salt was completely dissolved. Thereafter, the sample was filtrated (0.45 µm membrane filters; Merck, Millipore) and applied to an XK 16/20 column packed with a 20 ml β-cyclodextrin (β-CD) sepharose affinity resin and pre-equilibrated with 4 column volumes of 10 mM sodium acetate buffer, pH 5.5, with 500 mM NaCl at 1 ml/min [64]. After sample loading at 0.75 ml/min, the column was washed with eight column volumes of the above buffer at 1 ml/min. Bound proteins were eluted with four column volumes of 20 mM sodium acetate buffer, 7 mM β-CD, pH 5.5. The purification was carried out using an ÄKTA Avant chromatograph interfaced by UNICORN 5.0 control software (GE Healthcare).

The eluted sample was analyzed by SDS-PAGE using Novex NuPAGE^®^ 4–12 % Bis-Tris Gels (Invitrogen, Carlsbad, CA) in the XCell *Sure*Lock Mini-Cell system (invitrogen), according to manufacturer’s instructions and visualized by staining with InstantBlue solution (Expedeon, Cambridgeshire, UK). Spots were excised from the visually most prominent protein bands from the stained gel, washed in 300 µl 40 % ethanol at 50 °C for 15 min and in 100 µl 100 % ACN at room temperature for 10 min. The proteins in the gel were reduced with 50 µl 10 mM DTT in 100 mM NH_4_HCO_3_ at 56 °C for 45 min. Cysteine alkylation was performed by adding 100 µl 55 mM IAA in 100 mM NH_4_HCO_3_ and incubating at room temperature for 30 min in the dark. The gels were washed with 100 µl 50 % ACN, then 100 µl 100 % ACN and dried. The proteins in the gel were digested with 10 µl 12.5 ng/µl sequencing-grade modified trypsin (Promega) dissolved in 10 mM NH_4_HCO_3_ and incubated at 4 °C for 45 min, followed by addition of 10 µl 10 mM NH_4_HCO_3_ and incubation at 37 °C for 16 h. Samples (1 or 2 µl) were spotted directly onto a MTP AnchorChip target plate (Bruker Daltonics, Bremen, Germany), allowed to dry, and overlaid with 1 µl 0.5 µg/µl α-cyano-4-hydroxycinnamic acid (CHCA) matrix in 90 % ACN, 0.1 % TFA. The MS analyses were performed using an Ultraflex II MALDI-TOF/TOF MS (Bruker Daltonics). The obtained mass spectra were processed with FlexAnalysis and BioTools software both provided by the instrument manufacturer. Combination of MS and MS/MS data was used as input for databases searching for the spectra from MALDI-TOF/TOF using an in-house-licensed Mascot search engine (Matrix Science, London, UK). Proteins were identified using NCBInr database. The following parameters were set for searching: allowed global modification, carbamidomethyl cysteine; variable modification, oxidation of methionine; missed cleavages, 1; peptide tolerance, 80 ppm; MS/MS tolerance, ± 0.5 Da. The protein identification was considered valid if it matched more than two peptides and the significance threshold for protein identifications was *p* < 0.05.

### Identification of the consensus binding for the AmyR transcription factor

The AmyR consensus binding site sequence 5′-CGGN_8_CGG-3′ was identified by searching 1000 bp upstream of the start codon of the *A. nidulans* genes encoding *An*LPMO13A and *An*LPMO13B. The positions of the 5′ and 3′ ends of the AmyR binding site with putative translational start site as +1 were calculated.

## Additional files

10.1186/s13068-016-0604-0 Proteins identified in the secretome of *Aspergillus nidulans.* The table shows the MaxQuant output of identified proteins and the quantitative LFQ intensities associated with all replicates and conditions. Prediction of secretion was done as a combination of SignalP, Phobius and WoLF PSORT. CAZy annotation was done with dbCAN.
10.1186/s13068-016-0604-0 Heat map comparison of expression patterns of 312 secreted proteins detected after 3–5 days growth of *A. nidulans* on minimal media supplemented with: high-amylose (HA) maize, wheat, or pea starch. The colors in the heat map indicate the label-free quantification (LFQ) intensity reported by MaxQuant ranging from 2 × 10^6^ (light green) to 4 × 10^11^ (light red). Missing values were imputed from a normal distribution located at the quantification limit. Highlighted in green are AA13s, in purple—AA9s and AA11s, in blue—AA3s and AA7s, in red—hydrolytic enzymes associated with starch degradation, in gray—proteases, and in brown—cell wall-degrading enzymes. Secretion prediction is a combination of SignalP, Phobius, and WolfPSort where at least two algorithms had to agree.
10.1186/s13068-016-0604-0 A trending heat map of proteins predicted to be secreted during growth of *Aspergillus nidulans* on high-amylose (HA) maize, pea or wheat starch. Two main clusters are indicated in the graph, proteins with decreasing levels over time (I) and proteins with increasing levels over time (II). The intensities are z-score normalized per row, separately for each substrate, to emphasize the trend of protein amount during growth independent of total level and substrate-level differences. Proteins showing no change over time (ANOVA, permutation-based FDR >0.1) were removed from the plot. Missing values (protein level less than detection limit of mass spectrometer) are colored white. Highlighted in green are AA13s, in purple—AA9s and AA11s, in blue—AA3s and AA7s, in red—hydrolytic enzymes associated with starch degradation, in gray—proteases, and in brown—cell wall-degrading enzymes.
10.1186/s13068-016-0604-0 Comparison of detected CAZymes in the secretome of *Aspergillus nidulans* during growth on wheat, high-amylose maize and pea starch at day 3, 4 and 5.
10.1186/s13068-016-0604-0 **Table S3.** Top 20 detected proteins in the secretome of *Aspergillus nidulans* during growth on wheat starch at day 3, 4 and 5. **Table S4.** Top 20 detected proteins in the secretome of *Aspergillus nidulans* during growth on high-amylose maize starch at day 3, 4 and 5. **Table S5.** Top 20 detected proteins in the secretome of *Aspergillus nidulans* during growth on pea starch at day 3, 4 and 5.

## Abbreviations

AA: auxiliary activity enzyme
CAZyme: carbohydrate-active enzyme
CBM: carbohydrate-binding module
CDH: cellobiose dehydrogenase
β-CD: β-cyclodextrin
FGSC: fungal genetic stock center
GH: glycoside hydrolase
HA: high amylose
LC–MS/MS: liquid chromatography-tandem mass spectroscopy
LPMO: lytic polysaccharide monooxygenase
PL: pectin lyase
