# Supplementary figures and images for: Lytic polysaccharide monooxygenases and other oxidative enzymes are abundantly secreted by Aspergillus nidulans grown on different starches
Source: Biotechnol Biofuels. 2016 Sep 1;9(1):187. doi: 10.1186/s13068-016-0604-0 (PMC5007996; doi:10.1186/s13068-016-0604-0)

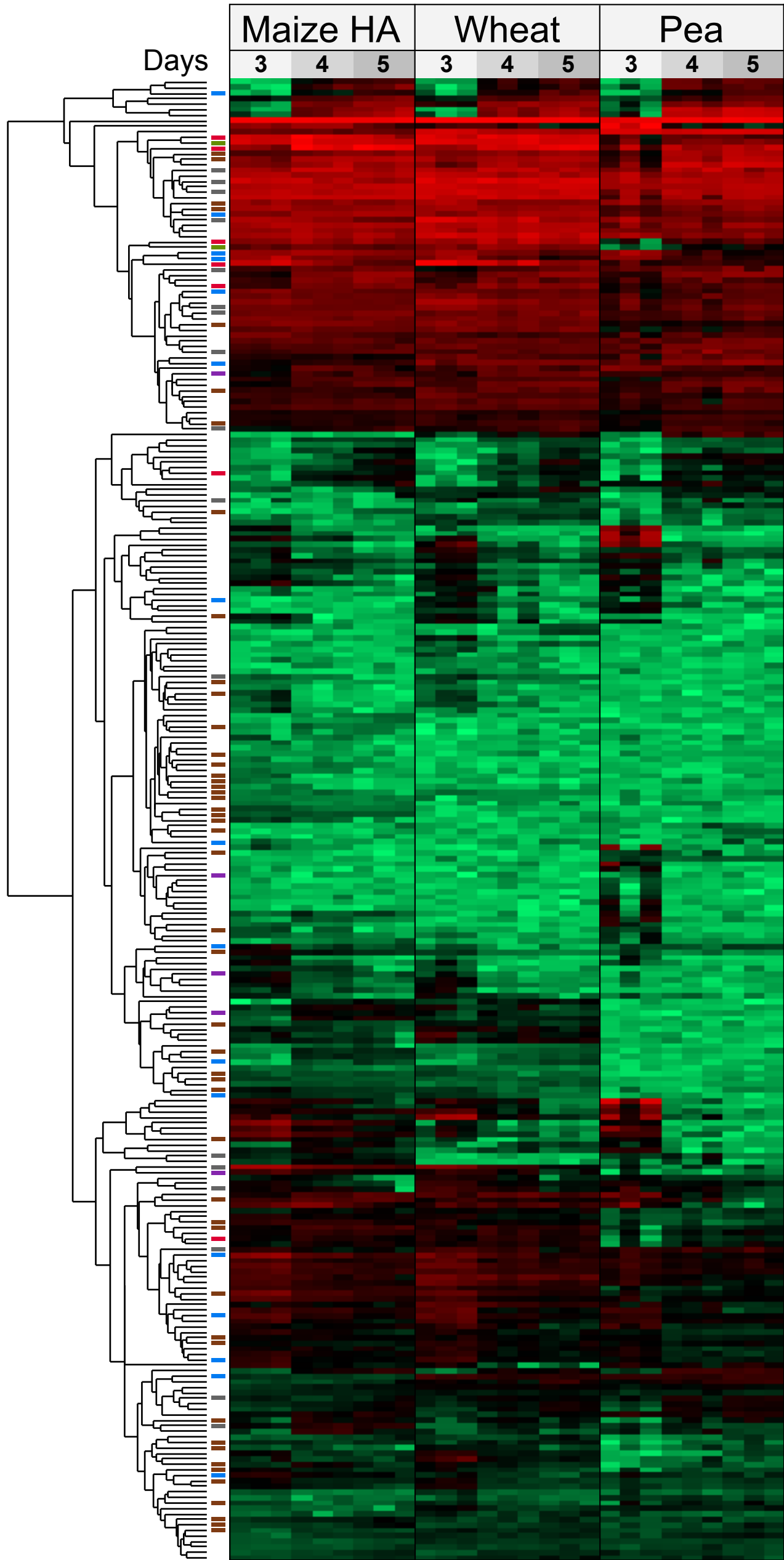

Supplement: Supplementary file 2 — 10.1186/s13068-016-0604-0 Heat map comparison of expression patterns of 312 secreted proteins detected after 3–5 days growth of A. nidulans on minimal media supplemented with: high-amylose (HA) maize, wheat, or pea starch. The colors in the heat map indicate the label-free quantification (LFQ) intensity reported by MaxQuant ranging from 2 × 10^6^ (light green) to 4 × 10^11^ (light red). Missing values were imputed from a normal distribution located at the quantification limit. Highlighted in green are AA13s, in purple—AA9s and AA11s, in blue—AA3s and AA7s, in red—hydrolytic enzymes associated with starch degradation, in gray—proteases, and in brown—cell wall-degrading enzymes. Secretion prediction is a combination of SignalP, Phobius, and WolfPSort where at least two algorithms had to agree. [file 13068_2016_604_MOESM2_ESM.pdf]

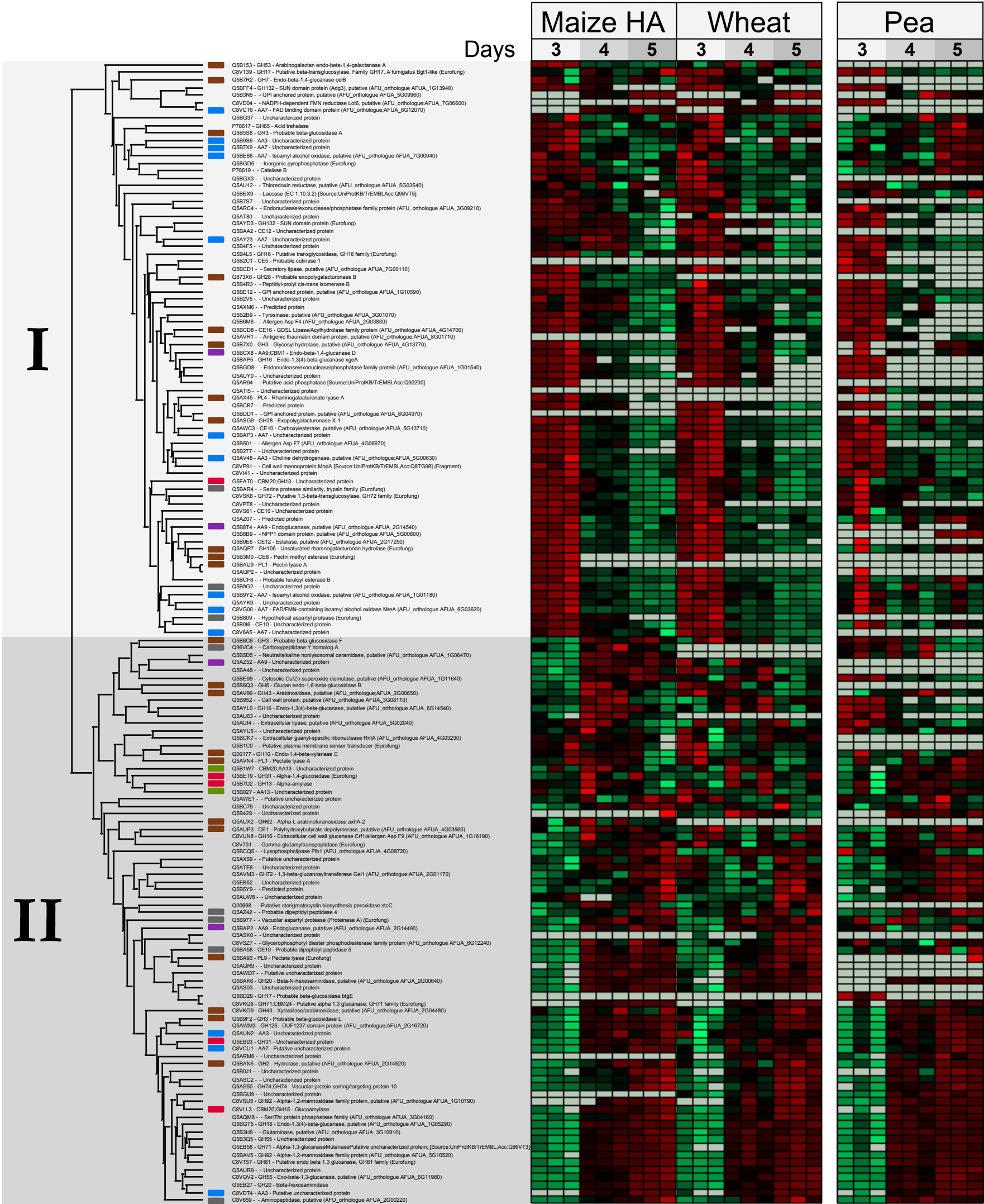

Supplement: Supplementary file 3 — 10.1186/s13068-016-0604-0 A trending heat map of proteins predicted to be secreted during growth of Aspergillus nidulans on high-amylose (HA) maize, pea or wheat starch. Two main clusters are indicated in the graph, proteins with decreasing levels over time (I) and proteins with increasing levels over time (II). The intensities are z-score normalized per row, separately for each substrate, to emphasize the trend of protein amount during growth independent of total level and substrate-level differences. Proteins showing no change over time (ANOVA, permutation-based FDR >0.1) were removed from the plot. Missing values (protein level less than detection limit of mass spectrometer) are colored white. Highlighted in green are AA13s, in purple—AA9s and AA11s, in blue—AA3s and AA7s, in red—hydrolytic enzymes associated with starch degradation, in gray—proteases, and in brown—cell wall-degrading enzymes. [file 13068_2016_604_MOESM3_ESM.pdf]
