# Supplementary material for: Lytic polysaccharide monooxygenases and other oxidative enzymes are abundantly secreted by Aspergillus nidulans grown on different starches
Source: Biotechnol Biofuels. 2016 Sep 1;9(1):187. doi: 10.1186/s13068-016-0604-0 (PMC5007996; doi:10.1186/s13068-016-0604-0)
Supplement: Supplementary file 4 — 10.1186/s13068-016-0604-0 Comparison of detected CAZymes in the secretome of Aspergillus nidulans during growth on wheat, high-amylose maize and pea starch at day 3, 4 and 5. [file 13068_2016_604_MOESM4_ESM.docx]

**Additional file 4: Supplementary Table S2**

**Supplementary Table S2**: Comparison of detected CAZymes in the secretome of *Aspergillus nidulans* during growth on wheat, high-amylose maize and pea starch at day 3, 4 and 5

| **CAZy family** |  | **Wheat starch** | | | **HA maize starch** | | | **Pea starch** | | |
| --- | --- | --- | --- | --- | --- | --- | --- | --- | --- | --- |
|  |  | *Day 3* | *Day 4* | *Day 5* | *Day 3* | *Day 4* | *Day 5* | *Day 3* | *Day 4* | *Day 5* |
| GH1 | PCW ^(a)^ | 1 | 1 | 1 | 1 | 1 | 1 | 0 | 1 | 1 |
| GH2 | PCW | 1 | 1 | 1 | 1 | 1 | 1 | 1 | 1 | 1 |
| GH3 | PCW | 5 | 5 | 5 | 5 | 5 | 5 | 4 | 4 | 5 |
| GH5 | PCW | 1 | 1 | 1 | 2 | 3 | 2 | 0 | 0 | 0 |
| GH6 | PCW | 1 | 1 | 1 | 1 | 1 | 1 | 0 | 0 | 0 |
| GH7 | PCW | 1 | 1 | 1 | 1 | 1 | 1 | 0 | 0 | 0 |
| GH10 | PCW | 1 | 1 | 1 | 1 | 1 | 1 | 1 | 1 | 1 |
| GH11 | PCW | 1 | 1 | 0 | 1 | 1 | 0 | 0 | 1 | 1 |
| GH13 | S | 3 | 3 | 3 | 3 | 3 | 3 | 3 | 3 | 3 |
| GH15 | S | 1 | 1 | 1 | 1 | 1 | 1 | 0 | 1 | 1 |
| GH16 |  | 6 | 6 | 5 | 6 | 6 | 5 | 6 | 4 | 4 |
| GH17 |  | 2 | 2 | 2 | 2 | 2 | 2 | 3 | 2 | 2 |
| GH20 |  | 1 | 2 | 2 | 2 | 2 | 2 | 1 | 2 | 2 |
| GH24 |  | 0 | 0 | 0 | 0 | 1 | 1 | 0 | 0 | 0 |
| GH25 |  | 3 | 3 | 2 | 3 | 3 | 1 | 3 | 3 | 2 |
| GH27 | PCW | 2 | 2 | 2 | 2 | 2 | 2 | 1 | 2 | 2 |
| GH28 | PCW | 1 | 1 | 1 | 2 | 2 | 2 | 1 | 1 | 1 |
| GH31 | S | 3 | 3 | 3 | 3 | 3 | 3 | 3 | 3 | 3 |
| GH35 | PCW | 0 | 0 | 0 | 1 | 1 | 0 | 0 | 1 | 1 |
| GH36 | PCW | 1 | 1 | 1 | 1 | 1 | 1 | 1 | 1 | 1 |
| GH43 | PCW | 4 | 4 | 4 | 4 | 4 | 3 | 3 | 4 | 3 |
| GH47 |  | 1 | 1 | 1 | 1 | 1 | 1 | 1 | 1 | 1 |
| GH53 | PCW | 1 | 1 | 1 | 1 | 1 | 1 | 0 | 0 | 0 |
| GH54 | PCW | 1 | 1 | 1 | 1 | 1 | 1 | 0 | 1 | 1 |
| GH55 |  | 2 | 2 | 2 | 2 | 2 | 2 | 2 | 2 | 2 |
| GH62 | PCW | 1 | 1 | 1 | 1 | 1 | 1 | 0 | 0 | 0 |
| GH63 |  | 1 | 1 | 1 | 0 | 0 | 1 | 1 | 1 | 1 |
| GH65 |  | 1 | 1 | 1 | 1 | 1 | 1 | 1 | 1 | 1 |
| GH71 |  | 0 | 2 | 2 | 1 | 2 | 2 | 1 | 2 | 2 |
| GH72 |  | 3 | 3 | 3 | 3 | 3 | 3 | 3 | 3 | 3 |
| GH74 | PCW | 1 | 1 | 1 | 1 | 1 | 1 | 1 | 1 | 1 |
| GH76 |  | 1 | 1 | 1 | 1 | 1 | 2 | 1 | 2 | 1 |
| GH81 |  | 1 | 1 | 1 | 1 | 1 | 1 | 1 | 1 | 1 |
| GH92 |  | 2 | 3 | 3 | 2 | 3 | 3 | 3 | 3 | 3 |
| GH93 | PCW | 1 | 1 | 1 | 1 | 1 | 1 | 1 | 1 | 1 |
| GH95 | PCW | 0 | 0 | 0 | 0 | 0 | 0 | 1 | 1 | 0 |
| GH105 | PCW | 1 | 1 | 1 | 1 | 1 | 1 | 1 | 1 | 1 |
| GH125 |  | 1 | 1 | 1 | 1 | 1 | 1 | 1 | 1 | 1 |
| GH132 |  | 2 | 2 | 0 | 2 | 2 | 1 | 2 | 2 | 0 |
| AA3 |  | 5 | 5 | 5 | 4 | 5 | 5 | 6 | 6 | 6 |
| AA7 |  | 8 | 10 | 10 | 9 | 10 | 9 | 7 | 8 | 8 |
| AA9 | PCW | 4 | 4 | 4 | 4 | 4 | 4 | 1 | 1 | 1 |
| AA11 |  | 0 | 0 | 0 | 0 | 0 | 0 | 1 | 0 | 0 |
| AA13 | S | 2 | 2 | 2 | 2 | 2 | 2 | 1 | 2 | 2 |
| PL1 | PCW | 2 | 2 | 1 | 3 | 1 | 1 | 1 | 1 | 1 |
| PL3 | PCW | 1 | 1 | 1 | 2 | 0 | 0 | 0 | 1 | 1 |
| PL4 | PCW | 0 | 0 | 0 | 1 | 1 | 0 | 0 | 0 | 0 |
| PL9 | PCW | 1 | 1 | 1 | 1 | 1 | 1 | 0 | 0 | 0 |
| CE1 | PCW | 3 | 3 | 3 | 3 | 3 | 3 | 2 | 3 | 3 |
| CE4 | PCW | 1 | 1 | 1 | 2 | 1 | 1 | 2 | 1 | 1 |
| CE5 |  | 0 | 0 | 0 | 0 | 0 | 0 | 1 | 0 | 0 |
| CE8 | PCW | 1 | 1 | 1 | 1 | 0 | 0 | 0 | 0 | 0 |
| CE10 |  | 7 | 6 | 6 | 7 | 6 | 6 | 6 | 6 | 6 |
| CE12 |  | 2 | 1 | 1 | 2 | 2 | 1 | 0 | 0 | 0 |
| CE16 | PCW | 1 | 0 | 1 | 1 | 1 | 1 | 1 | 0 | 0 |

The columns contain the numbers of enzymes detected from each CAZyme family. AA: Auxiliary Activity, CE: Carbohydrate Esterase, GH: Glycoside Hydrolase, GT: Glycoside Transferase, PL: Polysaccharide Lyase. (a): The families potentially active on plant cell walls (PCW) and starch (S) are indicated.
