# Supplementary material for: Lytic polysaccharide monooxygenases and other oxidative enzymes are abundantly secreted by Aspergillus nidulans grown on different starches
Source: Biotechnol Biofuels. 2016 Sep 1;9(1):187. doi: 10.1186/s13068-016-0604-0 (PMC5007996; doi:10.1186/s13068-016-0604-0)
Supplement: Supplementary file 5 — 10.1186/s13068-016-0604-0 Table S3. Top 20 detected proteins in the secretome of Aspergillus nidulans during growth on wheat starch at day 3, 4 and 5. Table S4. Top 20 detected proteins in the secretome of Aspergillus nidulans during growth on high-amylose maize starch at day 3, 4 and 5. Table S5. Top 20 detected proteins in the secretome of Aspergillus nidulans during growth on pea starch at day 3, 4 and 5. [file 13068_2016_604_MOESM5_ESM.docx]

**Additional file 5: Supplementary Tables S3 − S5**

**Supplementary Table S3**: Top 20 detected proteins in the secretome of *Aspergillus nidulans* during growth on wheat starch at day 3, 4 and 5

|  | **Protein** | | **Protein family** | | **Uniprot** |
| --- | --- | --- | --- | --- | --- |
| **Day 3** | |  | |  |  |
| 1 | Catalase B | |  | | P78619 |
| 2 | Uncharacterized protein | | GH13/CBM20 | | G5EAT0 |
| 3 | Serine protease similarity, trypsin family | |  | | Q5BAR4 |
| 4 | Uncharacterized protein | | AA13 | | Q5B027 |
| 5 | Thioredoxin reductase, putative | |  | | Q5AU12 |
| 6 | α-Amylase | | GH13 | | Q5B7U2 |
| 7 | Aminopeptidase Y, putative | |  | | Q5ATD5 |
| 8 | β-1,3-Glucanosyltransferase Gel1 | | GH72 | | Q5AVM3 |
| 9 | Alkaline protease 1 | |  | | Q00208 |
| 10 | Uncharacterized protein | |  | | C8V6E2 |
| 11 | Mannosyl-oligosaccharide α-1,2-mannosidase 1B | | GH47 | | Q5BF93 |
| 12 | Putative uncharacterized protein | |  | | Q5AWZ9 |
| 13 | Extracellular serine-rich protein, putative | |  | | Q5B926 |
| 14 | Arabinan endo-α-1,5-L-arabinosidase C | | GH43 | | Q5AUM3 |
| 15 | Uncharacterized protein | |  | | Q5AYU5 |
| 16 | Uncharacterized protein | | GH16 | | Q5AY11 |
| 17 | Neutral protease 2 homolog | |  | | Q5AUR8 |
| 18 | Probable β-glucosidase A | | GH3 | | Q5B5S8 |
| 19 | Cell wall mannoprotein MnpA | |  | | C8VP91 |
| 20 | Choline dehydrogenase, putative | | AA3 | | Q5AV48 |
| **Day 4** | | | | | |
| 1 | Catalase B | |  | | P78619 |
| 2 | α-Amylase | | GH13 | | Q5B7U2 |
| 3 | Uncharacterized protein | | AA13 | | Q5B027 |
| 4 | Aminopeptidase Y, putative | |  | | Q5ATD5 |
| 5 | Uncharacterized protein | | GH31 | | G5EB11 |
| 6 | Uncharacterized protein | |  | | C8V6E2 |
| 7 | Uncharacterized protein | | GH13/CBM20 | | G5EAT0 |
| 8 | Mannosyl-oligosaccharide α-1,2-mannosidase 1B | | GH47 | | Q5BF93 |
| 9 | Serine protease similarity, trypsin family | |  | | Q5BAR4 |
| 10 | Alkaline protease 1 | |  | | Q00208 |
| 11 | Neutral protease 2 homolog | |  | | Q5AUR8 |
| 12 | β-1,3-Glucanosyltransferase Gel1 | | GH72 | | Q5AVM3 |
| 13 | α-1,4-Glucosidase | | GH31 | | Q5BET9 |
| 14 | Extracellular serine-rich protein, putative | |  | | Q5B926 |
| 15 | Thioredoxin reductase, putative | |  | | Q5AU12 |
| 16 | Uncharacterized protein | |  | | Q5AYU5 |
| 17 | Putative uncharacterized protein | |  | | Q5AWZ9 |
| 18 | Putative uncharacterized protein | | AA7 | | C8VCU1 |
| 19 | Probable β-glucosidase L | | GH3 | | Q5B9F2 |
| 20 | Glutaminase A | |  | | C8VAK7 |
| **Day 5** | | | | | |
| 1 | Catalase B | |  | | P78619 |
| 2 | Uncharacterized protein | | GH31 | | G5EB11 |
| 3 | β-1,3-Glucanosyltransferase Gel1 | | GH72 | | Q5AVM3 |
| 4 | Uncharacterized protein | | AA13 | | Q5B027 |
| 5 | α-Amylase | | GH13 | | Q5B7U2 |
| 6 | Aminopeptidase Y, putative | |  | | Q5ATD5 |
| 7 | Mannosyl-oligosaccharide α-1,2-mannosidase 1B | | GH47 | | Q5BF93 |
| 8 | Alkaline protease 1 | |  | | Q00208 |
| 9 | Uncharacterized protein | |  | | C8V6E2 |
| 10 | Uncharacterized protein | |  | | Q5AYU5 |
| 11 | Extracellular serine-rich protein, putative | |  | | Q5B926 |
| 12 | α-1,4-Glucosidase | | GH31 | | Q5BET9 |
| 13 | Putative uncharacterized protein | |  | | Q5AWZ9 |
| 14 | Probable β-glucosidase L | | GH3 | | Q5B9F2 |
| 15 | Thioredoxin reductase, putative | |  | | Q5AU12 |
| 16 | Neutral protease 2 homolog | |  | | Q5AUR8 |
| 17 | Putative uncharacterized protein | | AA7 | | C8VCU1 |
| 18 | Uncharacterized protein | | GH55 | | Q5B3Q5 |
| 19 | Serine protease similarity, trypsin family | |  | | Q5BAR4 |
| 20 | Putative endo β-1,3-glucanase | | GH81 | | C8VT57 |

AA: Auxiliary Activity, GH: Glycoside Hydrolase.

**Supplementary Table S4**: Top 20 detected proteins in the secretome of *Aspergillus nidulans* during growth on high amylose maize starch at day 3, 4 and 5

|  | **Protein** | **Protein family** | **Uniprot** |
| --- | --- | --- | --- |
| **Day 3** | | | |
| 1 | Catalase B |  | P78619 |
| 2 | Uncharacterized protein | AA13 | Q5B027 |
| 3 | Uncharacterized protein | GH13/CBM20 | G5EAT0 |
| 4 | Aminopeptidase Y, putative |  | Q5ATD5 |
| 5 | α-Amylase | GH13 | Q5B7U2 |
| 6 | Mannosyl-oligosaccharide α-1,2-mannosidase 1B | GH47 | Q5BF93 |
| 7 | Alkaline protease 1 |  | Q00208 |
| 8 | Putative uncharacterized protein |  | Q5AWZ9 |
| 9 | β-1,3-Glucanosyltransferase Gel1 | GH72 | Q5AVM3 |
| 10 | Serine protease similarity, trypsin family |  | Q5BAR4 |
| 11 | Choline dehydrogenase, putative | AA3 | Q5AV48 |
| 12 | Extracellular serine-rich protein, putative |  | Q5B926 |
| 13 | Neutral protease 2 homolog |  | Q5AUR8 |
| 14 | Uncharacterized protein |  | C8V6E2 |
| 15 | Uncharacterized protein |  | Q5B9G2 |
| 16 | Probable β-glucosidase A | GH3 | Q5B5S8 |
| 17 | Arabinan endo-α-1,5-L-arabinosidase C | GH43 | Q5AUM3 |
| 18 | Uncharacterized protein |  | Q5AYU5 |
| 19 | Isoamyl alcohol oxidase, putative | AA7 | Q5B9Y2 |
| 20 | Uncharacterized protein | AA7 | Q5AY23 |
| **Day 4** | | | |
| 1 | Catalase B |  | P78619 |
| 2 | α-Amylase | GH13 | Q5B7U2 |
| 3 | Uncharacterized protein | GH31 | G5EB11 |
| 4 | Uncharacterized protein | AA13 | Q5B027 |
| 5 | Neutral protease 2 homolog |  | Q5AUR8 |
| 6 | Mannosyl-oligosaccharide α-1,2-mannosidase 1B | GH47 | Q5BF93 |
| 7 | Aminopeptidase Y, putative |  | Q5ATD5 |
| 8 | Alkaline protease 1 |  | Q00208 |
| 9 | β-1,3-Glucanosyltransferase Gel1 | GH72 | Q5AVM3 |
| 10 | Probable β-glucosidase L | GH3 | Q5B9F2 |
| 11 | Uncharacterized protein |  | C8V6E2 |
| 12 | Glutaminase A |  | C8VAK7 |
| 13 | Extracellular serine-rich protein, putative |  | Q5B926 |
| 14 | Putative uncharacterized protein | AA7 | C8VCU1 |
| 15 | Uncharacterized protein | GH13/CBM20 | G5EAT0 |
| 16 | Uncharacterized protein |  | Q5AYU5 |
| 17 | Uncharacterized protein | AA7 | Q5AY23 |
| 18 | Putative uncharacterized protein |  | Q5AWZ9 |
| 19 | α-1,4-Glucosidase | GH31 | Q5BET9 |
| 20 | Serine protease similarity, trypsin family |  | Q5BAR4 |
| **Day 5** | | | |
| 1 | Uncharacterized protein | GH31 | G5EB11 |
| 2 | Catalase B |  | P78619 |
| 3 | Uncharacterized protein | AA13 | Q5B027 |
| 4 | Mannosyl-oligosaccharide α-1,2-mannosidase 1B | GH47 | Q5BF93 |
| 5 | β-1,3-glucanosyltransferase Gel1 | GH72 | Q5AVM3 |
| 6 | α-Amylase | GH13 | Q5B7U2 |
| 7 | Alkaline protease 1 |  | Q00208 |
| 8 | Probable β-glucosidase L | GH3 | Q5B9F2 |
| 9 | Uncharacterized protein | GH55 | Q5B3Q5 |
| 10 | Putative uncharacterized protein |  | Q5AWZ9 |
| 11 | Putative uncharacterized protein | AA7 | C8VCU1 |
| 12 | Uncharacterized protein |  | C8V6E2 |
| 13 | Extracellular serine-rich protein, putative |  | Q5B926 |
| 14 | Aminopeptidase Y, putative |  | Q5ATD5 |
| 15 | Neutral protease 2 homolog |  | Q5AUR8 |
| 16 | Putative endo β 1,3 glucanase | GH81 | C8VT57 |
| 17 | Glutaminase A |  | C8VAK7 |
| 18 | β-Hexosaminidase | GH20 | G5EB27 |
| 19 | Probable β-glucosidase A | GH3 | Q5B5S8 |
| 20 | α-1,4-Glucosidase | GH31 | Q5BET9 |

AA: Auxiliary Activity, GH: Glycoside Hydrolase.

**Supplementary Table S5**: Top 20 detected proteins in the secretome of *Aspergillus nidulans* during growth on pea starch at day 3, 4 and 5

|  | | **Protein** | | **Protein family** | | **Uniprot** | |  |
| --- | --- | --- | --- | --- | --- | --- | --- | --- |
| **Day 3** | | | | | | | |  |
| 1 | | Uncharacterized protein | |  | | Q5B4F5 | | |
| 2 | | Catalase B | |  | | P78619 | | |
| 3 | | Thioredoxin reductase, putative | |  | | Q5AU12 | | |
| 4 | | SUN domain protein | | GH132 | | Q5AYD3 | | |
| 5 | | Serine protease similarity, trypsin family | |  | | Q5BAR4 | | |
| 6 | | Aminopeptidase Y, putative | |  | | Q5ATD5 | | |
| 7 | | Putative uncharacterized protein | |  | | Q5AWZ9 | | |
| 8 | | Uncharacterized protein | | AA7 | | Q5AY23 | | |
| 9 | | Uncharacterized protein | |  | | C8V6E2 | | |
| 10 | | Uncharacterized protein | | GH31 | | G5EB11 | | |
| 11 | | β-1,3-Glucanosyltransferase Gel1 | | GH72 | | Q5AVM3 | | |
| 12 | | Arabinan endo-α-1,5-L-arabinosidase C | | GH43 | | Q5AUM3 | | |
| 13 | | Allergen Asp F7 | |  | | Q5B501 | | |
| 14 | | Uncharacterized protein | |  | | Q5AT80 | | |
| 15 | | Alkaline protease 1 | |  | | Q00208 | | |
| 16 | | Choline dehydrogenase, putative | | AA3 | | Q5AV48 | | |
| 17 | | Uncharacterized protein | |  | | Q5B277 | | |
| 18 | | Cell wall mannoprotein MnpA | |  | | C8VP91 | | |
| 19 | | Mannosyl-oligosaccharide α-1,2-mannosidase 1B | | GH47 | | Q5BF93 | | |
| 20 | | Uncharacterized protein | | AA7 | | Q5AY23 | | |
| **Day 4** | | | | | | | |  |
| 1 | | Catalase B | |  | | P78619 | |  |
| 2 | | Thioredoxin reductase, putative | |  | | Q5AU12 | |  |
| 3 | | Glutaminase A | |  | | C8VAK7 | |  |
| 4 | | Aminopeptidase Y, putative | |  | | Q5ATD5 | |  |
| 5 | | Neutral protease 2 homolog | |  | | Q5AUR8 | |  |
| 6 | | Putative endo-β-1,3-glucanase | | GH81 | | C8VT57 | |  |
| 7 | | Uncharacterized protein | | GH55 | | Q5B3Q5 | |  |
| 8 | | Alkaline protease 1 | |  | | Q00208 | |  |
| 9 | | Uncharacterized protein | | GH31 | | G5EB11 | |  |
| 10 | | Putative uncharacterized protein | |  | | Q5AWZ9 | |  |
| 11 | | Hydrolase, putative | | GH2 | | Q5BAN5 | |  |
| 12 | | β-1,3-Glucanosyltransferase Gel1 | | GH72 | | Q5AVM3 | |  |
| 13 | | Mannosyl-oligosaccharide α-1,2-mannosidase 1B | | GH47 | | Q5BF93 | |  |
| 14 | | Probable β-glucosidase L | | GH3 | | Q5B9F2 | |  |
| 15 | | Arabinan endo-α-1,5-L-arabinosidase C | | GH43 | | Q5AUM3 | |  |
| 16 | | Hypothetical serine protease | |  | | C8VUL6 | |  |
| 17 | | Uncharacterized protein | | AA3 | | Q5AUN2 | |  |
| 18 | | Serine protease similarity, trypsin family | |  | | Q5BAR4 | |  |
| 19 | | Extracellular serine-rich protein, putative | |  | | Q5B926 | |  |
| 20 | | β-Hexosaminidase | | GH20 | | G5EB27 | |  |
| **Day 5** | | | | | | | |  |
| 1 | | Catalase B | |  | | P78619 | |  |
| 2 | | Thioredoxin reductase, putative | |  | | Q5AU12 | |  |
| 3 | | Glutaminase A | |  | | C8VAK7 | |  |
| 4 | | Uncharacterized protein | | GH55 | | Q5B3Q5 | |  |
| 5 | | Putative endo β-1,3-glucanase | | GH81 | | C8VT57 | |  |
| 6 | | Uncharacterized protein | | GH31 | | G5EB11 | |  |
| 7 | | Probable β-glucosidase L | | GH3 | | Q5B9F2 | |  |
| 8 | | Alkaline protease 1 | |  | | Q00208 | |  |
| 9 | | β-1,3-glucanosyltransferase Gel1 | | GH72 | | Q5AVM3 | |  |
| 10 | | Aminopeptidase Y, putative | |  | | Q5ATD5 | |  |
| 11 | | Mannosyl-oligosaccharide α-1,2-mannosidase 1B | | GH47 | | Q5BF93 | |  |
| 12 | | Hydrolase, putative | | GH2 | | Q5BAN5 | |  |
| 13 | | Putative uncharacterized protein | |  | | Q5AWZ9 | |  |
| 14 | | Extracellular serine-rich protein, putative | |  | | Q5B926 | |  |
| 15 | | Neutral protease 2 homolog | |  | | Q5AUR8 | |  |
| 16 | | Arabinan endo-α-1,5-L-arabinosidase C | | GH43 | | Q5AUM3 | |  |
| 17 | | Uncharacterized protein | | AA3 | | Q5AUN2 | |  |
| 18 | | β-Hexosaminidase | | GH20 | | G5EB27 | |  |
| 19 | | Probable β-glucosidase A | | GH3 | | Q5B5S8 | |  |
| 20 | | Uncharacterized protein | |  | | C8V6E2 | |  |

AA: Auxiliary Activity, GH: Glycoside Hydrolase.
